# Biotechnological Potential of Seaweeds from Bahia, Brazil: Metabolomic insights, Photoprotection and Antioxidant Activity

**DOI:** 10.1002/cbdv.202502708

**Published:** 2025-10-15

**Authors:** Keila Almeida Santana, Isadora de Jesus da Silva, Victor Pena Ribeiro, José Marcos de Castro Nunes, Hosana Maria Debonsi, Ian Castro‐Gamboa, Lorena Rigo Gaspar, Gustavo Souza dos Santos, Aníbal de Freitas Santos Júnior

**Affiliations:** ^1^ Department of Life Sciences State University of Bahia (UNEB) Salvador Bahia Brazil; ^2^ Department of Pharmaceutical Sciences, School of Pharmaceutical Sciences of Ribeirão Preto University of São Paulo (USP) Ribeirão Preto São Paulo Brazil; ^3^ Agricultural Research Service (ARS)—United States Department of Agriculture (USDA) Beltsville Maryland USA; ^4^ Department of Botany Institute of Biology Federal University of Bahia (UFBA) Salvador Bahia Brazil; ^5^ Department of Biomolecular Sciences, School of Pharmaceutical Sciences of Ribeirão Preto University of São Paulo (USP) Ribeirão Preto São Paulo Brazil; ^6^ Institute of Chemistry São Paulo State University (UNESP) Araraquara São Paulo Brazil

**Keywords:** antioxidants, mass spectrometry, natural products, sustainable chemistry, UV/Vis spectroscopy

## Abstract

Seaweeds have wide biotechnological applications. This study evaluated the chemical profile, photoprotective, and antioxidant potential of *Padina* sp., *Caulerpa sertularioides*, and *Solieria filiformis* collected in Bahia, Brazil. Chemical characterization by gas chromatography–mass spectrometry (GC–MS) and proton nuclear magnetic resonance (^1^H NMR) confirmed fucosterol in *Padina* sp., β‐sitosterol in *C. sertularioides*, and cholesterol in *S. filiformis* alongside fatty acids and aromatic metabolites; multivariate analyses supported species‐specific chemical patterns. The extracts exhibited ultraviolet (UV) absorption, in specific the UVA range, and were nonirritant in the Organization for Economic Co‐operation and Development (OECD) Test Guideline (TG) 491 assay. In human keratinocytes (HaCaT), *C. sertularioides* reduced UV‐induced reactive oxygen species (ROS) by 75%, whereas *Padina* sp. and *S. filiformis* achieved 42% reduction. Conversely, extracts exhibited photodegradation in the UV region and phototoxic potential in the OECD TG 432 (3T3 neutral red uptake, NRU) assay. Because these trials used complex crude extracts, future studies should optimize the extraction process, fractionate bioactive‐rich fractions, and isolate key compounds. These results highlight Brazilian algae as promising sources of new compounds and serve as a starting point for further investigations.

## Graphical abstract

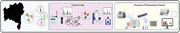

## 1 Introduction

Environmental changes, including the progressive ozone layer depletion, have intensified human exposure to solar ultraviolet (UV) radiation, raising major concerns regarding overall human health, as long‐term exposure can significantly compromise the structure and function of the skin [1]. UVB radiation (280–320 nm) is predominantly absorbed by the epidermis due to its shorter wavelength and the high concentration of chromophores in this layer, such as nucleic acids, proteins, and lipids, leading to direct DNA damage, inflammation, and erythema [2, 3]. Meanwhile, UVA radiation (320–400 nm), distinguished by its longer wavelength and lower energy, penetrates deeper, reaching the dermis, where it promotes collagen degradation, oxidative stress, and photoaging, mainly by affecting fibroblasts and components of the extracellular matrix [4]. Moreover, chronic exposure to both UVA and UVB radiation is associated with photocarcinogenesis [5].

Sunscreens have become essential tools for photoprotection due to their ability to absorb, reflect, or scatter the UV rays, mitigating the damage generated by rising levels of sun exposure [6]. However, several synthetic UV filters present limitations, such as photoinstability, allergenic potential, and environmental toxicity, particularly in marine ecosystems [7]. Among them, one example is avobenzone, that is, a widely used UVA filter, but it is photounstable and undergoes to rapid degradation upon exposure to sunlight, which compromises its protective performance and may lead to the formation of cytotoxic products [8]. Meanwhile, some studies report that organic UV filters, such as benzophenone‐3, can accumulate in marine environments at concentrations exceeding toxic thresholds, contributing to coral bleaching and other ecotoxicological effects [9].

Therefore, the search for sustainable bioactive compounds from marine sources has gained increasing prominence in the pharmaceutical, cosmetic, and nutraceutical industries [10]. To date, at least 11 drugs derived from marine organisms have been approved by regulatory agencies and are commercially available. Examples of marine‐derived compounds already used in the pharmaceutical industry include drugs for the treatment of cancer (*cytarabine*—Cytosar‐U, *trabectedin*—Yondelis) and viral infections (*vidarabine*—Vira‐A, and *iota‐carrageenan*—Carragelose), among others [11]. In the cosmetic field, several isolated marine substances have also demonstrated properties biological, such as porphyra‐334 and shinorine (Helioguard365), palythine, scytonemin, dieckol, triphlorethol‐A, and phycocyanin. In this context, macroalgae stand out for their ecological and biotechnological relevance, as they provide shelter and nourishment for marine fauna and produce a wide range of secondary metabolites. These compounds are closely linked to the algae's ability to adapt to adverse environmental conditions, such as UV radiation and fluctuations in salinity, temperature, and pH [12, 13].

Several studies have demonstrated the effectiveness of macroalgae extracts and their isolated compounds in various cosmetic applications [14]. Rangel et al. [15] evaluated Antarctic red algae extracts rich in mycosporine‐like amino acids (MAAs) that exhibited UV absorption comparable to synthetic UV filters, antioxidant potential, and low toxicity potential, highlighting their potential application in sunscreen and anti‐aging formulations. Similarly, Jordão et al. [16] identified photoprotective metabolites from an endophytic fungus associated with an Antarctic macroalga, which also showed high UVA/UVB absorption, photostability, antioxidant activity, and in vitro safety. These compounds were proven to act as natural UV filters and reactive oxygen species (ROS) scavengers. The ability of these bioactives to absorb UV radiation and neutralize free radicals, thus preventing photoaging, is usually attributed to polyphenols, carotenoids, fucoidans, and other bioactive compounds, which also contribute to skin hydration, brightness, and antimicrobial defense, expanding their potential use [17].

Despite the potential of the Brazilian coastline, which stretches for over 7000 km, limitations to the exploration of marine macroalgae persist, such as the lack of commercial‐scale cultivation and the concentration of research on specific applications [18, 19]. In a national survey, Santos et al. [10] identified 71 species with reported biotechnological applications. Antioxidant activity was the most common, appearing in 29% of the studies, whereas photoprotective activity was mentioned in only 6%, highlighting the still underexplored nature of this area. Harb et al. [29] evaluated the antioxidant activity of macroalgae collected in the Northeast (Paraíba) and Southeast (Espírito Santo) regions, reporting that northeastern species stood out [20]. This result was attributed to regional environmental variations. Complementarily, Schmitz et al. [31] observed differences in the photoprotective indices of algae collected in different regions of the Brazilian coast, indicating that regional environmental factors directly influence the bioactivity of these species.

Seaweeds from the coast of Bahia, Brazil, have been gaining prominence due to their biotechnological potential. A well‐recognized application is in renewable energy production, where algal biomass is processed into pellets with a high calorific value (8.82–20.18 MJ/kg), comparable to that of terrestrial sources like sugarcane bagasse, thereby contributing to reduced environmental impacts [21]. Furthermore, Bahia is the home to one of the most diverse macroalgal floras in the country, harboring approximately 53% of all marine species identified in Brazil. This region is well recognized as a biodiversity hotspot and stands out not only for its taxonomic richness but also for its remarkable genetic diversity [22, 34]. Martins et al. [23] analyzed 148 samples, a brown macroalgae, from 12 Brazilian coastal sites and identified two distinct genetic lineages, with populations in the north, mainly in Bahia, exhibiting haplotype and nucleotide diversity 14.6 and 15.5 times greater, respectively, than those in the south. Similar patterns were noted by Faria et al. [22] in other species, including the red alga *Gracilariopsis tenuifrons*, which likewise shows clear genetic structure impacted by coastal geographic factors. These studies highlight the northeastern region of Brazil as a reservoir of genetic diversity and reinforce the strategic importance of Bahia for the conservation and bioprospecting of native macroalgae.

Despite these advances, in‐depth chemical characterization and biological validation of macroalgae from Bahia remain largely unexplored. However, several species have demonstrated the ability to produce bioactive compounds, such as lipids, carotenoids, phycocolloids, and lectins, which reinforces their potential for pharmaceutical and cosmetic applications [10]. In this context, Schmitz et al. [20] identified and evaluated the photoprotective potential of the brown macroalgae from Bahia, *Sargassum vulgare* and *Dictyota mertensii*, collected in Barra Grande, located on the Maraú Peninsula, in the south of the state. Although these species showed high UV absorption rates, suggesting the presence of phenolic and carotenoid compounds with antioxidant and photoprotective activity, data are still limited. To date, this was the only study identified on the subject. Therefore, this study focuses on three tropical macroalgae from Bahia, collected on the north coast, near the capital Salvador, belonging to different phyla: *Padina* sp., *Caulerpa sertularioides*, and *Solieria filiformis*, which were selected according to their structural diversity and biotechnological potential.

The brown macroalgae *Padina* sp. (Ochrophyta) occurs along the Brazilian coast, particularly in Bahia, in the regions of All Saints Bay and Porto Seguro, where it grows in shallow intertidal and subtidal zones, attached to rocky substrates [24, 25]. *Padina* species exhibit ecological plasticity and play essential roles in coastal ecosystems [26]. Chemically, their extracts are rich in compounds such as phlorotannins, flavonoids, fucoidans, and carotenoids. These secondary metabolites are associated with reported biological effects, such as antioxidant, photoprotective, antimicrobial, antiproliferative, anti‐inflammatory, and enzyme inhibitory activities [27, 28]. An example is fucoidan, a polysaccharide containing fucose, other minor sugars, and sulfate and uronic acid residues, which contribute to its bioactivity. These structural characteristics were confirmed by spectroscopic analyses [29, 30].

*C. sertularioides* (Chlorophyta) is a green macroalga distributed in tropical marine environments, with occurrences along the Brazilian coastline, especially in the Northeast region. It exhibits rapid growth and high adaptability to environmental stressors [31, 32, 33]. In Bahia, it was recorded for the first time on rocky mesolitoral coasts and sublittoral zones in Salvador in 1991 by Nunes and collaborators. The species has a diverse bioactive profile, notably rich in sulfated polysaccharides, flavonoids, phenolic acids, terpenoids, sterols, and saponins, which are associated with antioxidant, antibacterial, anti‐inflammatory, antibiofilm, antimicrobial, immunostimulant, antitumor, and antidiabetic properties [34]. Additionally, it is a source of caulerpin, which stands out for its multiple biological activities. Nursidika et al. [35] also reported antifungal activity against *Candida albicans*, where it acts as a fungistatic agent, compromising fungal cell integrity. These findings highlight the multifunctional potential of caulerpin, as well as the various bioactive compounds of seaweed.

*S. filiformis* (Rhodophyta) is a tropical red macroalgae widely distributed worldwide, present in warm coastal regions of the Atlantic. It is a native and well‐established species on the Brazilian coast, especially in Bahia. It can be found on rocky substrates in coastal areas and attaches itself through rhizoids [36]. *S. filiformis* extracts exhibit diverse biological activities associated with their chemical composition, including sulfated polysaccharides, lectins, carotenoids, and phycobiliproteins [37]. Chaves et al. [38] analyzed the SfL 1 isoform of the lectin from *S. filiformis*, including its recombinant form (rSfL‐1), and confirmed that both promote wound healing by reducing the inflammatory response and stimulating rapid and intense collagen deposition, favoring tissue repair and skin regeneration. Furthermore, Liu et al. [39] isolated a new polyketide, named solieritide A, from the genus *Solieria*, along with six other secondary metabolites. Among these, four showed structural similarity to sescandeline, a compound previously isolated by Santos [40], which demonstrated photoprotective and antioxidant potential comparable to commercial UV filters, without presenting. Given this chemical and functional diversity, *S. filiformis* also stands out as a promising source of natural compounds with multiple applications.

Based on this framework, the present study investigates the biotechnological potential of three tropical macroalgae species from Bahia—*S. filiformis*, *Padina* sp., and *C. sertularioides—*through an integrated assessment of their chemical composition, photoprotective, and antioxidant potential. By combining advanced analytical techniques such as gas chromatography–mass spectrometry (GC–MS) and proton nuclear magnetic resonance spectroscopy (^1^H NMR), as well as biological assays using alternative methods to animal use to evaluate the efficacy and toxicity potential of these algae extracts. The analysis included UV–Vis absorption, in vitro phototoxicity potential, in vitro quantification of UVA‐induced ROS in human keratinocyte (HaCaT), and short‐term eye irritation potential [41, 42]. This work not only advances the chemical and biological characterization of underexplored marine resources from Bahia but also explores the bioactivity of extracts with antioxidant and photoprotective potential applications as sustainable and multifunctional ingredients. These findings are expected to contribute for the development of effective, safe, and environmentally friendly alternatives to conventional synthetic ingredients, particularly in the cosmetic industry.

## 2 Results and Discussion

The chemical characterization of extracts from the macroalgae *S. filiformis*, *Padina* sp., and *C. sertularioides*, collected off the coast of Bahia, was assessed using GC–MS, ^1^H NMR, and molecular networking. This integrative approach enabled the identification of major metabolites and the exploration of their structural relationships. Furthermore, we evaluated their photoprotective, antioxidant, and safety properties. For this purpose, we applied alternative methods to animal experimentation, in accordance with international guidelines.

### 2.1 Chemical Profile

Metabolomics is the science that studies the set of low‐molecular‐mass metabolites in organisms, with the goal of understanding how they react to different conditions [43]. GC–MS analysis offers high sensitivity for volatile and semivolatile metabolites; however, annotations using this technique in crude extracts are inherently putative and subject to limitations due to spectral similarity, coelution, and analytical artifacts [44]. ^1^H NMR analysis, on the other hand, provides global spectral profiles of nonvolatile and polar compounds [45]. The combination of GC–MS and ^1^H NMR techniques allowed for a more comprehensive identification of the compounds present in macroalgae extracts, overcoming the limitations associated with the use of each method alone. This integration contributed to increased analytical robustness and enabled a more in‐depth characterization of natural extracts [43]. The mass spectral data of the main metabolites detected are presented in Table S1.

From this perspective, Parchemin et al. [46] investigated chemical variations in two *Asparagopsis* species using a multiblock metabolomics approach combining ^1^H NMR and HS–SPME–GC–MS. Samples collected from five locations over a 2‐year period were analyzed using biphasic extraction, enabling the identification of polar, nonpolar, and volatile compounds, including chemotaxonomic markers and metabolites associated with environmental variation. Despite these differences, the antibacterial activity of the extracts remained consistent over time. Kanai et al. [47] combined ^1^H NMR and GC–MS data using orthogonal projections to latent structures (OPLS) modeling to identify marker metabolites in complex extracts of raw (GR) and processed (PGR) ginger. The integration of these techniques overcame limitations of NMR analysis alone, allowing the identification of α‐r‐curcumen as a differential marker between GR and PGR. Therefore, this approach reduced laborious isolation steps and demonstrated potential for metabolomics‐based quality control. The use of GC–MS and ^1^H NMR techniques, combined with modern data extraction and analysis methods, has expanded the ability to characterize metabolites present in complex macroalgae extracts with greater precision and comprehensiveness [23, 48]. The innovative application of these tools to tropical species from understudied regions, such as the coast of Bahia, constitutes a contribution to the field and serves as a central motivation for this study.

Considering the chemical complexity of natural extracts and the dimensionality of data generated by GC–MS and ^1^H NMR techniques, the application of chemometric tools is essential for data interpretation. The use of multivariate analysis, through exploratory methods, such as principal component analysis (PCA), and supervised methods, such as partial least squares discriminant analysis (PLS‐DA), allows the identification of hidden patterns, the discrimination of samples based on their metabolic profiles, and the selection of key metabolites for further investigation [49]. Meunier et al. [50] applied an integrated approach combining molecular networks (MS^2^), PLS modeling, which focuses on identifying correlations between chemical variables and continuous biological responses, and ^13^C NMR dereplication to investigate bioactive metabolites in *Garcinia parvifolia* peel extract. They identified an abundance of prenylated xanthones and, using NMR‐based dereplication, were able to annotate potentially active compounds within complex mixtures. This approach proved effective in guiding the identification of bioactive substances without requiring prior isolation, reinforcing the value of multivariate methods for studying complex natural matrices such as macroalgae.

PCA was applied to investigate the metabolic variability among extracts of the three macroalgae. This technique revealed a clustering pattern by phylum, with *C. sertularioides* (Chlorophyta), *Padina* sp. (Ochrophyta), and *S. filiformis* (Rhodophyta) forming distinct groups (in material of Supporting Information), indicating specific chemical profiles of each lineage. Principal Component 1 (PC1), responsible for 21% of the total variance, was primarily responsible for separating *C. sertularioides* from *Padina* sp. Principal component 2 (PC2), which explained 14.8% of the variance, distinguished *S. filiformis* from the other species. Palanisamy et al. [51] analyzed the chemical diversity of three macroalgae from different phyla: *Ulva reticulata* (Chlorophyta), *Sargassum wightii* (Ochrophyta), and *Gracilaria* sp. (Rhodophyta). Using GC–MS and PCA, more than 30 metabolites were detected, which were shown to be the main metabolites responsible for the differentiation between species. Thus, these data corroborate the findings, indicating that PCA allowed clear discrimination between phyla and not only supports the understanding of taxonomic and ecological differences between species but also provides a basis for future biotechnological applications (Figure S1).

The observed pattern was reinforced by PLS‐DA, which demonstrated an even more pronounced separation between the taxonomic groups. The first two components of the model, Component 1 (17.2%) and Component 2 (16.9%), explain the significant total variance, reflecting differences in metabolite composition between the species. The model achieved 80% classification accuracy, confirming its ability to distinguish samples based on their chemical profiles. The high coefficient of determination (*R* ^2^ > 0.99) indicates that the model explains the variation in the data used to construct it. The *Q* ^2^ value (0.26), obtained through cross‐validation, predicts the species or phylum that a sample belongs to, based on the metabolites present in the extract. Furthermore, variable importance in projection (VIP) scores were obtained, allowing the identification of the metabolites that most influence the distinction between groups, functioning as chemical markers. Values greater than 1 indicate a significant contribution to group separation, where the chromatographic peaks highlighted by the PLS‐DA model reveal specific chemical signatures associated with each macroalgal species or phylum (Figure S1).

In *Padina* sp. (Ochrophyta), fucosterol emerged as the main discriminant in the multivariate model, being a sterol, a structural isomer of fucosterol, which differs in the position of the double bond in the core of the steroid ring. These metabolites are commonly found in brown macroalgae and are characterized by conjugated ring systems, responsible for their antioxidant and UV‐absorbing properties [52]. *Padina pavonica* and other brown algae, including *Ecklonia cava*, *Fucus vesiculosus*, and *Sargassum muticum*, are known to be rich in phlorotannins, unique polyphenolic compounds derived from phloroglucinol, reinforcing the chemical affinity of *Padina* species with this class of bioactive secondary metabolites [53]. Therefore, this finding is in agreement with the study by Henri et al. [28] and Nour et al. [29], who reported similar chemical compositions (Figure S1).

The characteristics with the highest VIP values are related to the substances 9,12,15‐octadecatrienoic acid, (*Z*,*Z*,*Z*), β‐sitosterol, and bicyclo[4.1.0]heptane, 7‐methylene‐, from the alga *C. sertularioides* (Chlorophyta), classified as fatty acids, sterols, and terpenes, respectively, and are chemical groups recognized for their abundance in green macroalgae. Zeng et al. [54] reported the presence of fatty acids (palmitic acid, oleic acid, and nonanoic acid) and terpenes (betulinic acid, oleanolic acid, ursolic acid, and myrcene) in the green alga *Ulva prolifera*. Teco‐Bravo et al. [55] also reported the presence of fatty acids and terpenes, as well as the sterol campesterol, in *Chlorella saccharophila*, which corroborates the results of the present study (Figure S1).

*S. filiformis* (Rhodophyta) was characterized by the presence of compounds such as *n*‐hexadecanoic acid and cholesterol, which were highly concentrated in this group, demonstrating the presence of phylum‐specific metabolites such as fatty acids and sterols, as previously reported. Akbary et al. [56] investigated the red alga *Ahnfeltiopsis pygmaea*, which showed a high content of saturated fatty acids, a low content of monounsaturated fatty acids, and a moderate content of polyunsaturated fatty acids. It is notable for the high presence of arachidonic acid and eicosapentaenoic acid, in addition to sitostanol as the major sterol, which is consistent with the predominant components in our species (Figure S1).

Serviere‐Zaragoza et al. [39] conducted a study in Baja California Sur, Mexico, and observed that each macroalgal phylum has a characteristic sterol profile. In red algae, cholesterol predominates, followed by *t*‐dehydrosterol and brassicasterol; in brown algae, fucosterol is the main component, accompanied by campesterol and isofucosterol. However, in green algae, isofucosterol predominates, with smaller proportions of cholesterol, fucosterol, brassicasterol, or norcholesterol. These patterns reinforce the usefulness of sterol profiles as taxonomic markers among different groups of macroalgae, although other variations may occur.

**FIGURE 1:**
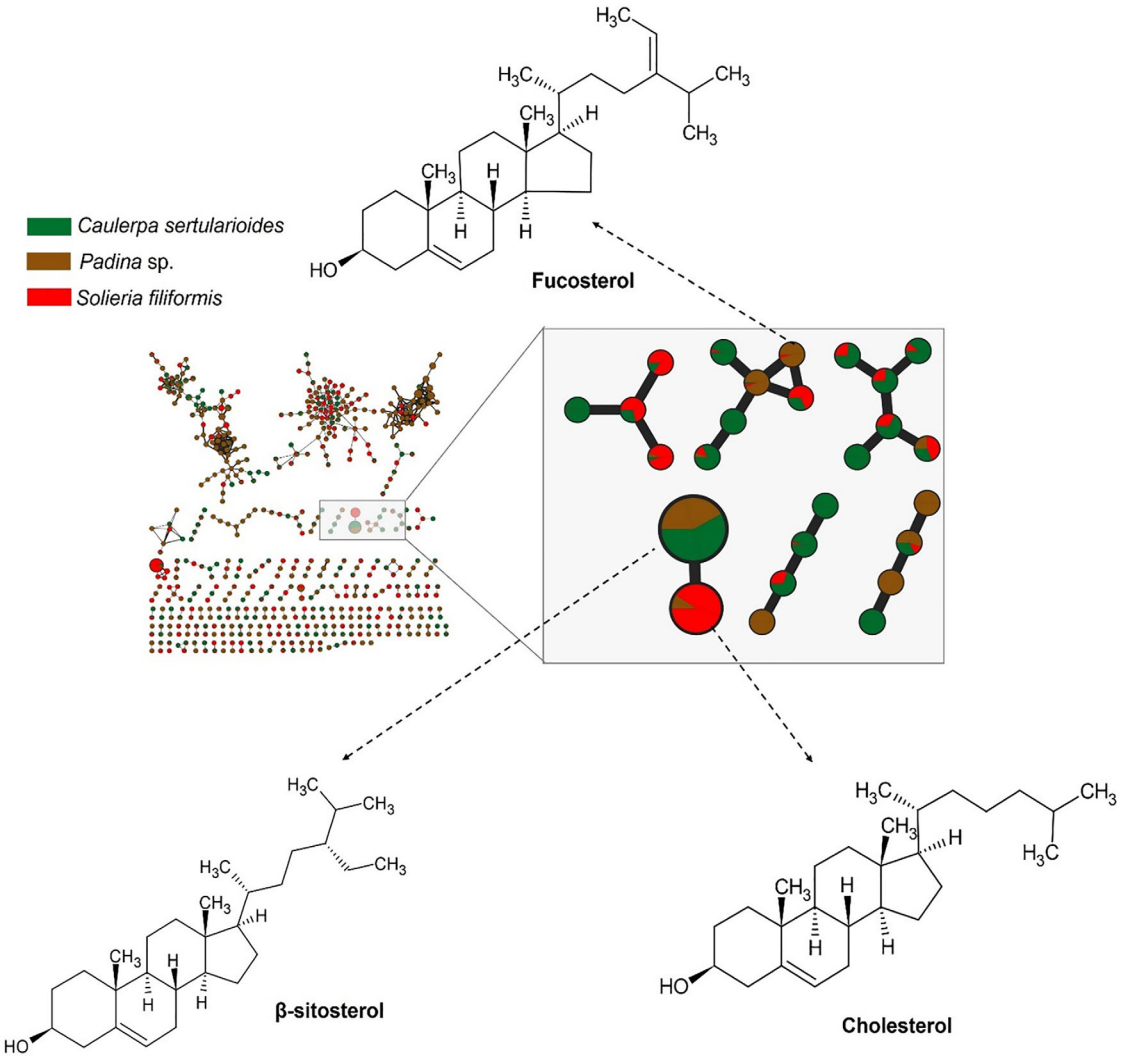
Molecular network of sterols obtained by GC–MS from extracts seaweeds. Recurrent compounds, such as fucosterol, β‐sitosterol, and cholesterol, were highlighted, whose distribution profiles follow taxonomic patterns described for macroalgae. Node colors indicate species distribution. Edge thickness and color are proportional to the cosine score calculated by GNPS, representing spectral similarity between connected nodes. *Source*: Image created by the authors.

**FIGURE 2:**
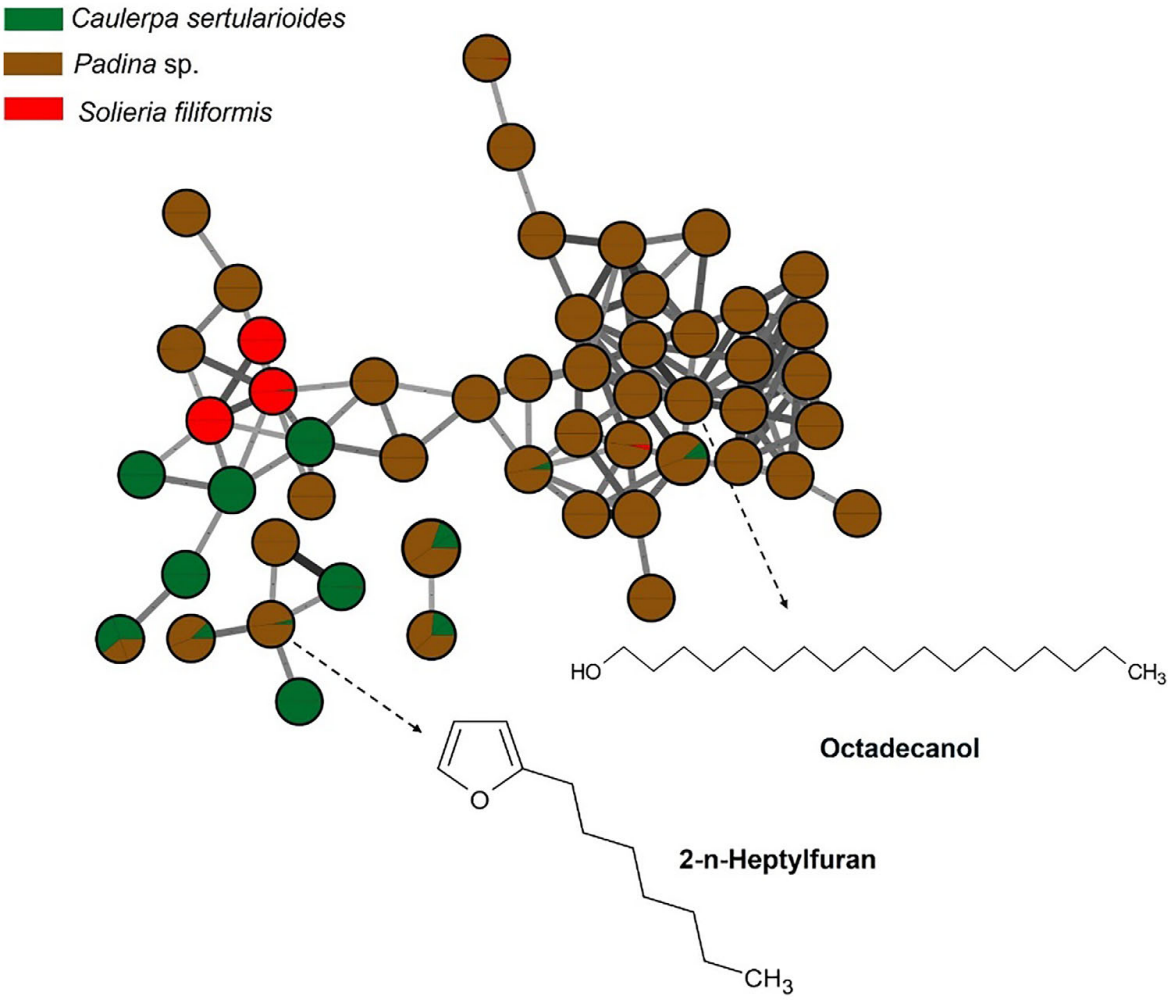
Major metabolites of the algae *Padina* sp. obtained from GC–MS analysis and represented in a molecular network, in which they are grouped by spectral similarity. Edge thickness and color are proportional to the cosine score, representing spectral similarity between connected nodes. *Source*: Image created by the authors.

This distribution pattern is illustrated in Figure 1, which depicts the molecular network obtained from GC–MS analysis, comprising 457 nodes and 531 edges distributed across 105 clusters. The network reveals a moderate degree of chemical diversity and modular organization among the metabolites detected in the analyzed macroalgae. Figure 2 also highlights annotated and connected compounds, such as fucosterol, β‐sitosterol, and cholesterol, whose recurrence and distribution reflect the evolutionary, chemical, and biosynthetic divergences among algal phyla, consistent with the multivariate analysis data [49].

The brown alga *Padina* sp. presented a chemical profile consistent with metabolites characteristic of the phylum Ochrophyta, with compounds such as fucosterol, represented in Figure 2. The presence of fucosterol, the main sterol present in brown algae, reinforces the bioactive potential of the species, as this compound has neuroprotective, hypocholesterolemic, and immunomodulatory properties, with recognized applications in the cosmeceutical and nutraceutical industries [57].

Other compounds present in the brown seaweed extract was octadecanol, identified by GC–MS analysis (Figure 2). Octadecanol, a long‐chain fatty alcohol, is known for its emollient, antimicrobial, and antioxidant properties and is widely used in cosmetic formulations due to its ability to improve skin barrier function and reduce transepidermal water loss [57]. Gallic acid, a well‐known phenolic compound, exhibits strong antioxidant and anti‐inflammatory activity, and its presence is consistent with the occurrence of phlorotannin‐like metabolites in brown algae [58].

**FIGURE 3:**
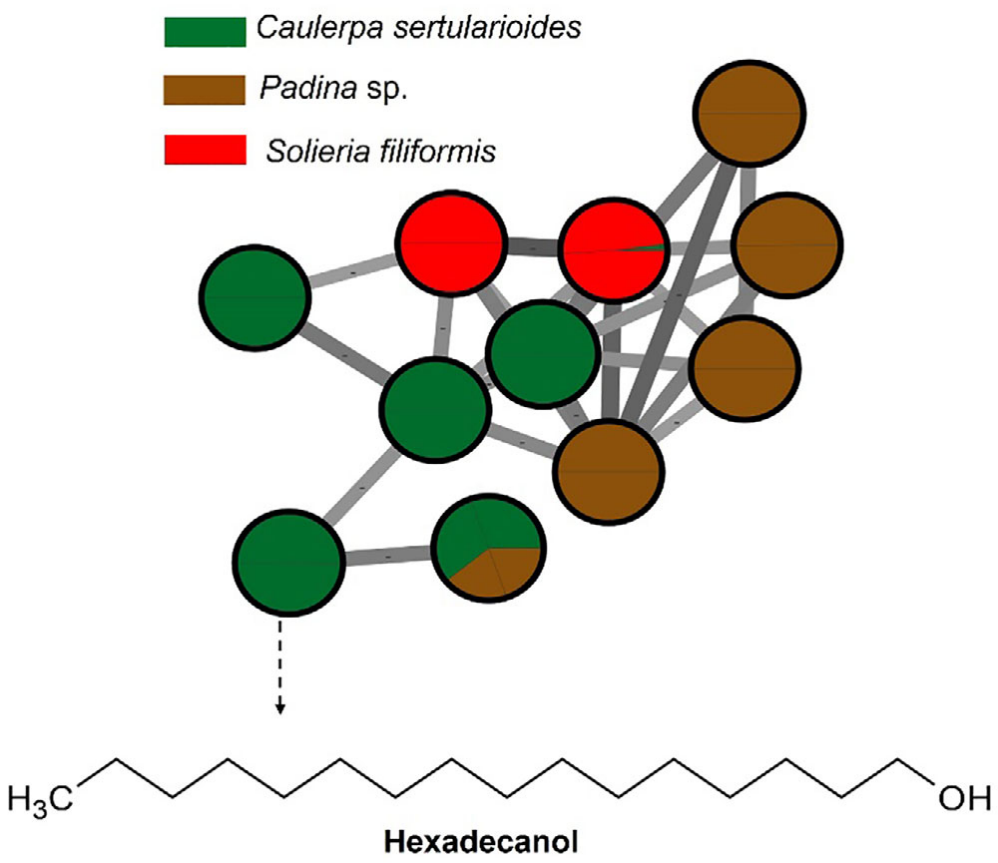
Major metabolites of the red alga *Caulerpa sertularioides* obtained from GC–MS analysis and represented in a molecular network, where nodes are grouped by spectral similarity. Edge thickness and color are proportional to the cosine score, representing the degree of spectral similarity between connected nodes. *Source*: Image created by the authors.

The green alga *C. sertularioides* presented as its main marker β‐sitosterol, a phytosterol with recognized anti‐inflammatory, antioxidant, hypocholesterolemic, and immunomodulatory effects (Figure 3). An interesting compound was hexadecanol (or cetyl alcohol), a long‐chain fatty alcohol with emollient, antimicrobial, and humectant properties, widely used in cosmetic formulations for its ability to reinforce the skin barrier and reduce transepidermal water loss [59, 60]. Additionally, caldariacetal alcohol tetraacetate, a terpenoid with an “H”‐shaped structure, stands out for suggesting metabolic plasticity in the algae, possibly associated with adaptation to adverse environmental conditions, such as high salinity, intense solar radiation, and temperature variations, typical of the northern coast of Bahia [19, 61]. These findings complement the results of Anjali et al. [62], who demonstrated the antioxidant, antibacterial, and immunomodulatory properties of sulfated polysaccharides from *C. sertularioides*, reinforcing the biochemical versatility of the species through the production of hydrophilic and lipophilic compounds with potential cosmetic, pharmaceutical, and nutraceutical applications; the compounds are represented in Figure 3.

**FIGURE 4:**
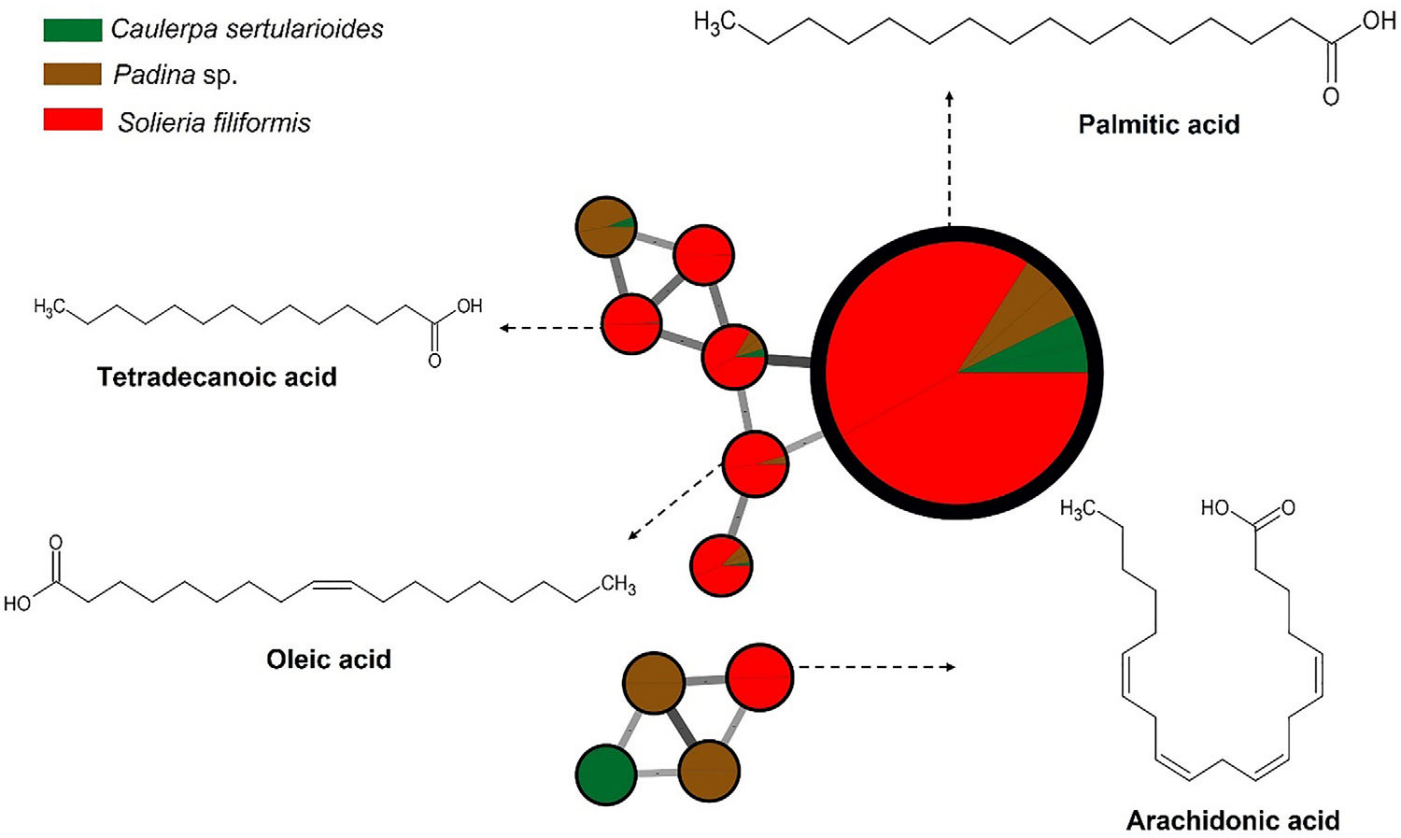
Major metabolites of the red alga *Solieria filiformis* obtained from GC–MS analysis and represented in a molecular network, where nodes are grouped by spectral similarity. Edge thickness and color are proportional to the cosine score, representing the degree of spectral similarity between connected nodes. *Source*: Image created by the authors.

The red alga *S. filiformis* presented a chemical profile characterized by the presence of fatty acids such as palmitic, oleic, tetradecanoic, and arachidonic acids, as well as lipophilic compound, such as cholesterol, as observed in GC–MS analyses. Carpena et al. [63] reported that fatty acids similar to those noted in the samples contribute to anti‐inflammatory and cardioprotective effects. Additionally, cholesterol was noted, which is frequently reported in Rhodophyta as the dominant sterol. However, other sterols such as desmosterol, in addition to small amounts of sitosterol and fucosterol, may also be present. Although sterols predominate per phylum, there is considerable overlap between groups, and none of these compounds is completely exclusive to a single lineage [64, 65]. The compounds are illustrated in Figures 1 and 4.

Although valuable, GC–MS has limitations in detecting polar, thermolabile, or high molecular weight compounds in complex extracts, such as sulfated polysaccharides, MAAs, phlorotannins, peptides, and carotenoids [66, 67, 68]. These molecules often possess polar functional groups and bulky structures that hinder volatilization and degrade at high analysis temperatures [69]. Many of these metabolites, however, play key ecological roles in photoprotection, gaining relevance as promising bioactives. MAAs absorb UVA radiation, whereas sulfated polysaccharides form protective antioxidant barriers. Likewise, phlorotannins, carotenoids, and peptides mitigate oxidative stress caused by sun exposure [27, 70]. Therefore, ^1^H NMR spectroscopy was used as a complementary method, allowing broader detection of such metabolites and enhancing the chemical characterization of the extracts.

Spectral data obtained by ^1^H NMR spectroscopy allowed the identification of key chemical shift changes associated with specific metabolite classes, reinforcing the annotations obtained by GC–MS and molecular networks. Turupadang [71] used a similar strategy combining GNPS‐based molecular networks with ^1^H NMR liquid chromatography–tandem MS (LC–MS/MS) data to prioritize macroalgae extracts from West Timor. This approach allowed the selection of three extracts containing characteristic signals in the 3.5–5.0 ppm region, typically attributed to oxymethine protons and glycosidic structures.

Liu et al. [72] used ^1^H NMR to analyze 100 samples of *Lonicerae japonicae* flos and *Lonicerae* flos, revealing signals that allowed distinguishing the species, cultivation modes, and processing methods. The generated spectra were subjected to chemometric analysis, with accuracy (>95%), identifying differential markers such as macrantoidin A and B, dipsacoside B, secoxyloganin, secologanoside, and sweroside. These examples reinforce the relevance of integrated approaches in the chemical characterization of complex natural extracts, such as those from the marine environment.

**FIGURE 5:**
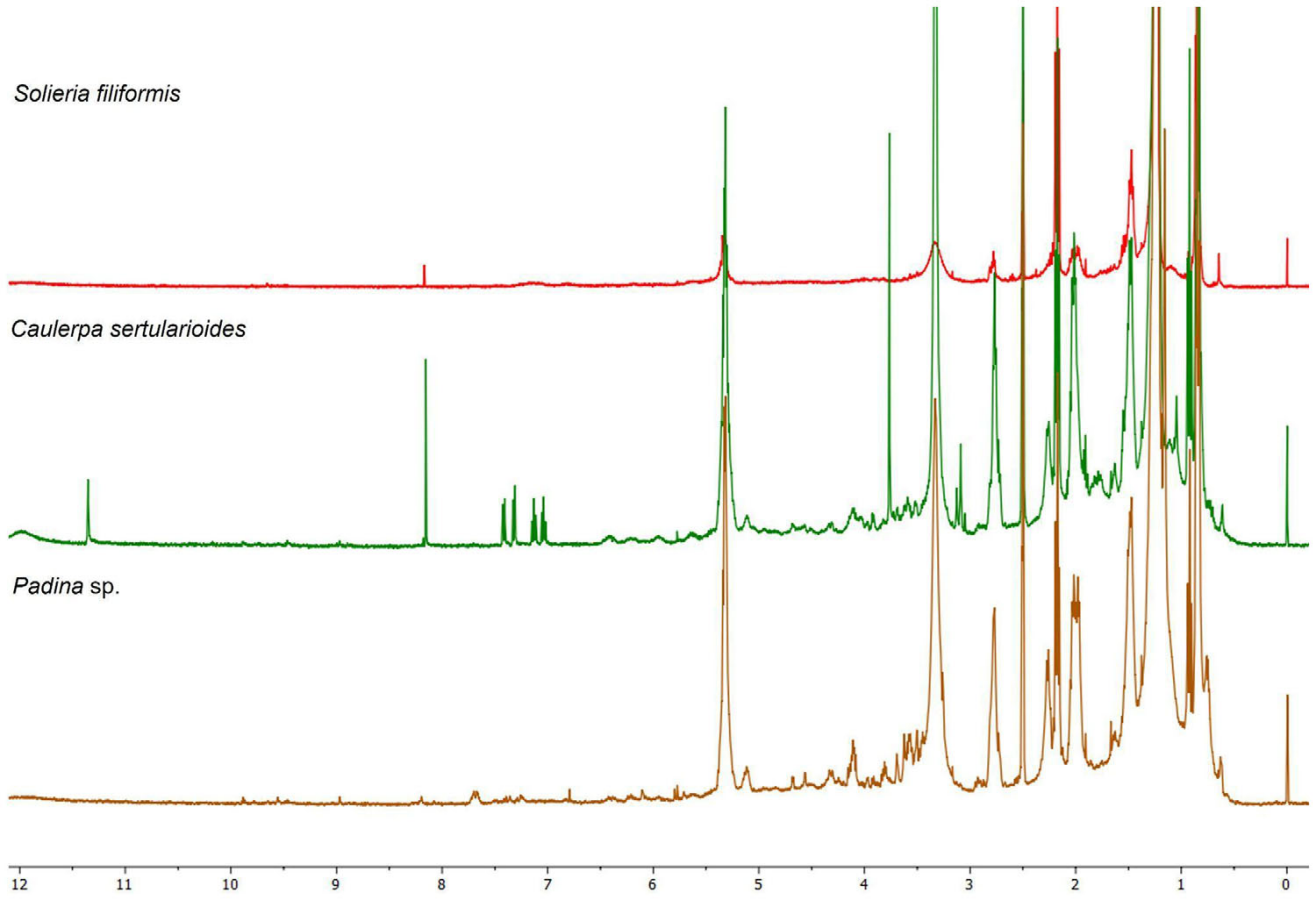
^1^H NMR spectra of the ethyl acetate extracts from tropical seaweed. Spectra were in DMSO‐d_6_ at 400 MHz and 25°C. Characteristic signals are observed in three main regions: Aliphatic (*δ* 0.5–3.0 ppm), carbohydrate/glycosidic (*δ* 3.0–5.5 ppm), and aromatic/olefinic (*δ* 6.0–9.0 ppm) (*δ* 10.0–12.0 ppm) are indicative of aldehydic protons, suggesting the presence of conjugated aldehydes or aromatic systems with extended resonance. The peak at *δ* 2.50 ppm corresponds to the residual solvent signal. *Source*: Image created by the authors.

The ^1^H NMR spectrum of *Padina* sp. presented in Figure 5 indicates the regions of the spectrum and the multiplicities corresponding to distinct hydrogen environments. The extract revealed intense signals in the aliphatic region (*δ* 0.8–2.5 ppm), consistent with the presence of saturated and unsaturated fatty acids. It also demonstrated methyl and methylene groups of compounds previously described in brown macroalgae, suggesting the presence of compounds such as palmitic acid and oleic acid, consistent with the established GC–MS profile. The region between *δ* 3.6 and 4.0 ppm presented a broad, defined signal, characteristic of mannitol, a polyol common in the cell walls of algae of the *Padina* genus. This compound is frequently identified in crude extracts prepared by nonselective methods, and its high‐intensity detection corroborates the presence of water‐soluble metabolites with a structural function [28].

In contrast, signals in the *δ* region of 5.5–6.5 ppm, attributed to aromatic protons of phenolic compounds such as phlorotannins, were weak or absent, suggesting a low abundance of these constituents in the crude extract or their possible association with macromolecular structures that hinder detection by ^1^H NMR. Nour et al. [29] demonstrated that selective purification by liquid–liquid partitioning or solid‐phase extraction can concentrate phlorotannins and reveal signals in this region, such as those related to phloroglucinol (*δ *∼ 5.98 ppm) and polyhydroxylated aromatic rings, with the seaweed *P. pavonica*. Thus, the spectral profile observed in *Padina* sp. reflects a composition with sugars and fatty acids, with modest levels of phenolic compounds, consistent with the phytochemical profile already established [28].

The ^1^H NMR spectrum of *C. sertularioides* (Figure 5) complemented the GC–MS findings by revealing signals across a broader polarity range, including metabolites that are generally not detected by GC due to low volatility or thermal lability [66]. Intense resonances were observed in the aliphatic region (*δ* 0.5–3.0 ppm), consistent with the presence of long‐chain fatty acids, fatty alcohols, and sterols, such as hexadecanol and sitosterol, already identified in the chromatographic analysis. Additional signals between *δ* 3.0 and 5.5 ppm suggest the presence of hydroxylated or glycosylated protons, indicative of secondary alcohols, phenols, or sugar residues derived from sulfated polysaccharides [62]. Although discreet, the signals in the aromatic/olefinic region (*δ* 5.5–8.0 ppm) point to the possible presence of flavonoids or bromophenols, metabolites related to antioxidant and photoprotective activities in green macroalgae. Additionally, a minor signal observed between *δ* 10.0 and 12.0 ppm is characteristic of aldehydic protons in highly conjugated systems, which may contribute to extended resonance and, consequently, to UV absorption behavior [27, 60, 70].

The ^1^H NMR spectrum of *S. filiformis* (Figure 5) exhibited the following signals complementary to those identified by GC–MS. The spectrum obtained exhibited a predominantly polar pattern, with a single intense and well‐defined signal in the aromatic/olefinic region (*δ* 6.5–8.5 ppm), characteristic of highly substituted aromatic rings, such as halogenated phenols. This profile is consistent with the presence of bromophenols, which are widely reported in Rhodophyta species [64, 70]. These compounds, although invisible to GC–MS analysis due to their low volatility and thermal lability, were evidenced by NMR as relevant contributors to the chemical identity of the species [66]. This aromatic signature was corroborated by chemometric data, with variables in this range represented by the VIP score in the PLS‐DA analysis, suggesting that they act as discriminating markers for *S. filiformis* in relation to the other macroalgae analyzed.

Furthermore, the region between *δ* 3.0 and 5.5 ppm presented moderate‐intensity signals, indicative of protons on carbons neighboring hydroxyl and amino groups, often associated with glycosylated structures, lectins, or MAAs [37, 67]. These findings are consistent with the detection of total carbohydrates and R‐phycoerythrin reported by Abreu et al. [38] and Sousa et al. [37], as well as with the presence of the lectin isolated by Abreu et al. [55], all studies conducted with *S. filiformis*. The low signal intensity in the aliphatic range (*δ* 0.8–2.0 ppm) suggests a lower relative abundance of neutral lipids, such as saturated and unsaturated fatty acids, a finding consistent with the composition observed in chromatographic analyses, which revealed lower lipophilic complexity compared to other species analyzed [63]. This integrated approach not only expanded the scope of the chemical characterization of *S. filiformis* but also reinforced its metabolic uniqueness compared to other species.

*C. sertularioides* presented less chemical complexity compared to *Padina* sp. and S. *filiformis*, as evidenced by the simplicity of the spectrum and the partial overlap in the PLS‐DA plots. Nevertheless, the presence of signals characteristic of aliphatic and aromatic compounds corroborates literature reports on the species’ bioactive repertoire, such as sulfated polysaccharides, phenolic compounds, and flavonoids [60]. The integrated application of GC–MS and ^1^H NMR, therefore, was decisive in expanding the chemical characterization of this species, highlighting its potential as a source of multifunctional ingredients with moisturizing, antioxidant, and photoprotective properties for cosmetic use (Figure S2).

These findings reflect the chemodiversity of macroalgae across different phyla, highlighting their potential for biotechnological applications. The growing demand for sustainable and multifunctional ingredients has driven the search for natural alternatives to UV filters and synthetic antioxidants, with a focus on marine resources [10]. Chemical characterization of extracts is essential because the metabolite profile determines biological activity, safety, and applicability in foods, pharmaceuticals, and cosmetics, for example [73, 74]. Therefore, we performed chemical characterization tests to identify and quantify key compounds, followed by biological assays to confirm the efficacy and biocompatibility of the extracts.

### 2.2 Evaluation of Photoprotective Properties

In recent decades, national and international legislation restricting or prohibiting the use of animals in cosmetics testing has significantly boosted the development and validation of alternative in vitro and in silico methods. The European Union pioneered a total ban on animal testing for cosmetics and their ingredients in 2013, as established by Regulation No. 1223/2009 [75]. Since then, countries, such as India, Israel, Norway, Australia, and Mexico, have also adopted similar restrictions. In Brazil, regulatory progress has intensified in recent years. Law No. 11794/2008 established the 3Rs principles (reduction, refinement, and replacement) and created the National Council for the Control of Animal Experimentation (CONCEA), responsible for recognizing validated alternative methods [76]. More recently, Law No. 15183/2025 definitively prohibited the use of animals in testing personal care products, cosmetics, perfumes, and their ingredients, reinforcing the country's commitment to ethical and sustainable practices [77]. Accordingly, validated alternative methods were employed in the present work to evaluate the irritation, phototoxicity, and antioxidant potential of the tropical seaweeds.

### 2.3 UV Absorption Spectra

**FIGURE 6:**
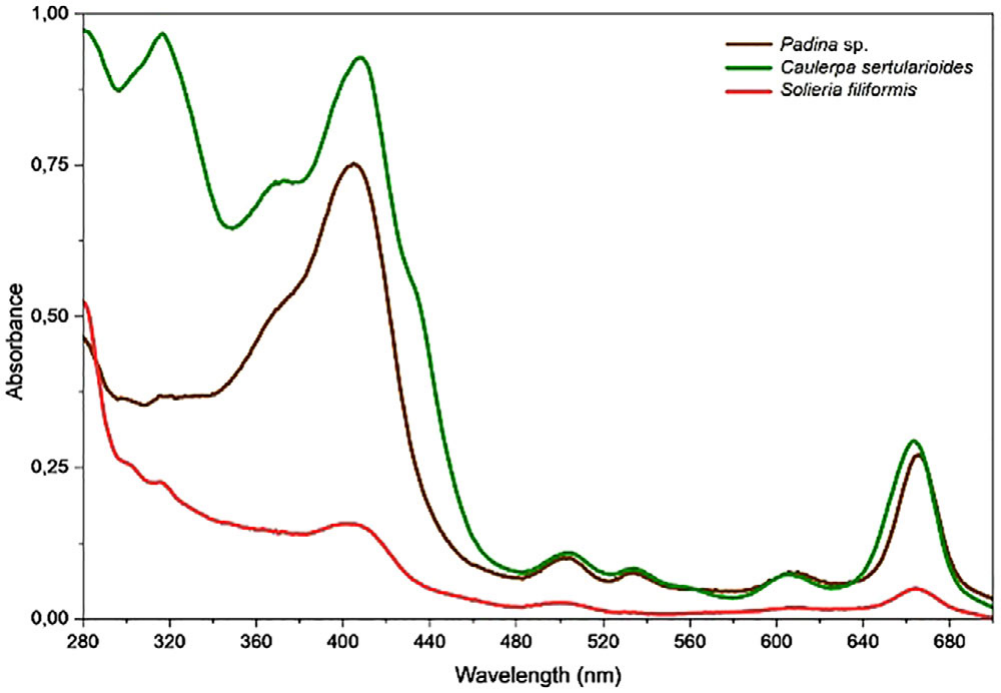
Absorption spectra of the *Padina* sp., *Solieria filiformis*, and *Caulerpa sertularioides* crude extracts. Representative curves obtained from triplicate samples in methanol solution, 100 µg/mL. *Source*: Image created by the authors.

The evaluation of the spectral absorbance across UVB, UVA, and visible‐light regions not only enables a comparative assessment but also highlights the photoprotective potential of algae extracts, such as that obtained from *Padina* sp., *S. filiformis*, and *C. sertularioides* [20]. The crude extracts of all the three algae exhibited significant absorption in the entire UV region, including high absorption in the UVA range (320–400 nm) (Figure 6). By examining how their extracts interact with specific energy bands of the electromagnetic spectrum, it was possible to infer the presence of bioactive compounds that might act against solar exposition. Nevertheless, further investigations, particularly regarding their efficacy and safety profiles, were required before considering their application as UV filters [1].

The UV–Vis absorption spectrum of the *Padina* sp. crude extract (Figure 6) showed a band with absorption between 400 and 430 nm, indicating optical activity in the UV and visible regions. This spectral profile is consistent with the presence of compounds containing conjugated systems capable of absorbing light in different bands of the electromagnetic spectrum, a hypothesis reinforced by GC–MS data, in which metabolites with the potential to act both in radiation absorption and ROS neutralization were noted, such as octadecanol and gallic acid [28, 57, 78]. Although the signals attributed to phlorotannins were discrete in the ^1^H NMR spectra, studies such as that described by Nour et al. [29] demonstrated that the dosage of phlorotannins varies according to the extraction method used; techniques such as liquid–liquid partitioning tend to reveal more complex structures, whereas other methods tend to miss some compounds.

*Padina* sp. also showed a significant absorbance in the visible‐light region, as *C. sertularioides*. Brown algae usually exhibit a more complex pigment matrix, where chlorophyll a coexists with chlorophyll c, pheophytin a, and the xanthophyll, such as fucoxanthin, the latter not only conferring their characteristic color but also serving as a potent antioxidant and bioactive compound [79, 80]. Tavares et al. [80] isolated fucoxanthin from *Desmarestia anceps* and demonstrated that its incorporation at 0.5% (w/v) into a sunscreen formulation increased the overall UV absorption spectrum by 72%, significantly enhanced antioxidant effects, and showed no phototoxicity in reconstructed human skin (RHS). These findings underscore the potential of pigments from green and brown algae as multifunctional ingredients for broad‐spectrum photoprotective formulations.

The UV–Vis absorption spectrum of *C. sertularioides* (Figure 6) showed well‐defined bands in the UV and visible regions, also consistent with the chemical profile determined in this study. The presence of metabolites annotated by GC–MS indicates the occurrence of high conjugated structures capable of absorbing radiation in this spectral range, while the signals detected in the ^1^H NMR spectra suggest additional compounds with photoprotective potential [27, 62, 70]. The observed absorption in the visible‐light region is also consistent with the presence of pigments such as chlorophylls (A and B) and carotenoids, typically found in species of the genus *Caulerpa*, which, in addition to acting in light capture, contribute to protection against photooxidative damage [81, 82]. Yalçin et al. [79] observed that in green algae, such as *Caulerpa racemosa* var. *cylindracea* and *Codium fragile*, chlorophyll A and chlorophyll B were the predominant pigments, accompanied by significant amounts of carotenoids such as β‐carotene, violaxanthin, and siphonaxanthin. The authors also evaluated the antioxidant capacity of these algae. Thus, the spectral characteristics of *C. sertularioides* corroborate and expand the metabolic evidence obtained by chromatographic and spectroscopic analyses.

The UV–Vis absorption spectrum of the crude extract of *S. filiformis* (Figure 6) showed discrete but continuous bands throughout the UV and visible regions, indicating a moderate optical absorption capacity. This profile corroborates with the chemical composition of the species revealed by GC–MS and ^1^H NMR analyses, which indicated the presence of halogenated aromatic and phenolic compounds, such as bromophenols, with conjugated structures capable of absorbing radiation in different bands of the electromagnetic spectrum [66, 70]. Furthermore, signals in the *δ* region 3.0–5.5 ppm in the NMR spectra suggest the presence of MAAs, known to act as natural UV filters in red algae, and absorbance in the UVB regions [67]. Thus, the spectral signature of *S. filiformis* reflects a chemical composition compatible with photoprotection, although less pronounced than those observed for *Padina* sp. and *C. sertularioides*.

Therefore, the UV–Vis spectra of the three macroalgae revealed distinct photoprotective potentials, consistent with their chemical compositions. In *Padina* sp., the absorption indicated the presence of lipophilic compounds and conjugated aromatic structures; *C. sertularioides* stood out for its intense absorption in all the UV and visible‐light regions, attributed to the presence of high conjugated compounds and pigments, such as carotenoids and chlorophylls. However, *S. filiformis* presented a more discreet profile but was consistent with the presence of bromophenols and MAAs. These data preliminarily indicate the photoprotective and antioxidant potential of these extracts.

### 2.4 Photostability Assessment

**FIGURE 7:**
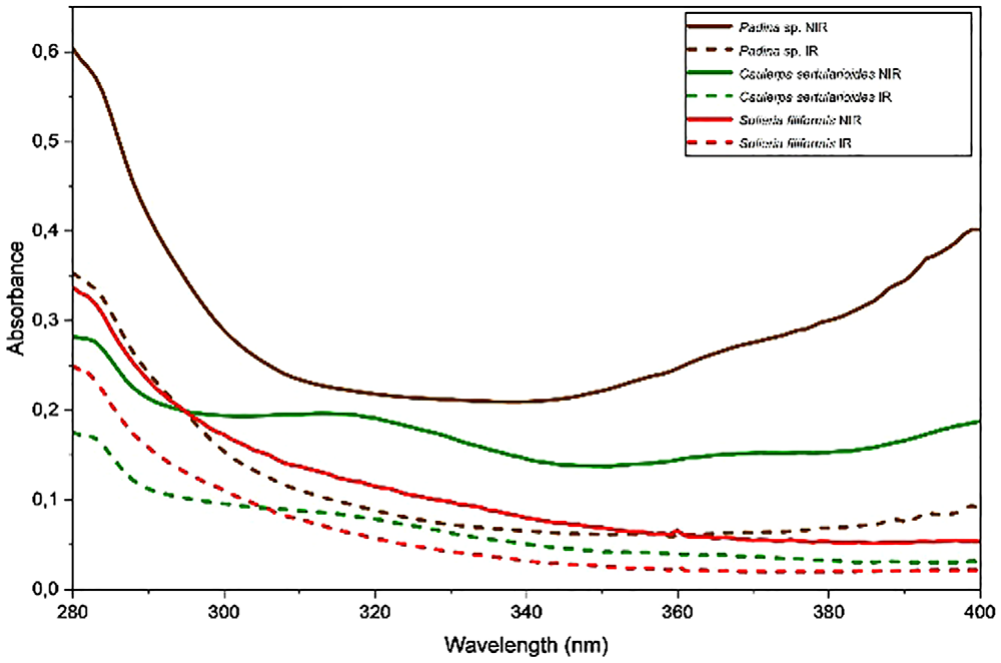
Absorption spectra of the seaweed extracts photostability: nonirradiated (Nirr—solid line) and irradiated with 9 J/cm^2^ UVA dose (irr—dashed line). Representative curves obtained from triplicate samples in isopropanol solution, 100 µg/mL (three independent experiments). *Source*: Image created by the authors.

The photodegradation rates revealed that *C. sertularioides* and *Padina* sp. exhibited similar levels of photodegradation, approximately 47% in the UVA region and 72% in the UVB region. In contrast, *S. filiformis* showed lower photodegradation, with 35% in the UVA region and 60% in the UVB region (Figure 7 and Table 1). The assessment of photostability involves monitoring changes in the absorbance following exposure to UV radiation. Photoprotective compounds typically contain chromophoric groups capable of absorbing energy within the UV/Vis spectrum. Upon exposure to radiation, these chromophores absorb energy and transition to an excited state. The absorbed energy is subsequently dissipated through various processes, including fluorescence, phosphorescence, fragmentation, isomerization, interactions with surrounding molecules, and the generation of free radicals. However, during these pathways, molecular degradation may occur, compromising the protective efficacy of such compounds [15, 83].

**TABLE 1:** Absorbance reduction is represented by the area under the curve of the irradiated samples (UVA dose of 9 J/cm^2^) compared to nonirradiated samples (considered 100% in the UVA and UVB range) of the algae extracts.

Sample	Photodegradation (%)	
	UVA	UVB
*Padina* sp.	73.90 ± 3.03	46.08 ± 1.94
*Caulerpa sertularioides*	72.45 ± 1.48	49.75 ± 1.97
*Solieria filiformis*	60.47 ± 0.07	35.21 ± 2.01

*Note*: Values are presented as mean ± standard deviation (SD), (*n* = 3, three independent experiments).

Several widely used UV‐filtering compounds are prone to degradation upon UV exposure, which can diminish their photoprotective efficacy [7]. For example, avobenzone, despite its widespread use in sunscreens due to its UVA absorption, undergoes significant photodegradation in the UV region. The combination of avobenzone with photostabilizing agents, such as octocrylene, can enhance its stability [84]. Similarly, fucoxanthin was reported in the literature to be initially considered photounstable; however, when incorporated into sunscreen formulations, it exhibited photostability, with degradation remaining below 20%, thereby demonstrating a UV radiation‐enhancing effect [80].

Although the extracts showed high photodegradation levels, according to the International Conference on Harmonisation (ICH) guideline “Stability Testing: Photostability Testing of New Drug Substances and Products Q1B,” while no specific limits for acceptable photodegradation are defined, confirmatory studies must ensure that UV exposure does not induce chemical changes affecting the safety or efficacy of the substance or product. Functional evaluations through spectrophotometric scanning, quantification of UV filters degradation via high‐performance liquid chromatography (HPLC), and identification of degradation products using MS are key strategies to elucidate the mechanisms involved when the substance is exposed to UV radiation. Nonetheless, phototoxicity testing remains a primary requirement to verify the safety of photounstable compounds and to meet international regulatory standards for photostabilized sunscreen formulations [85]. Therefore, the evaluating of the phototoxicity potential is essential to determine whether the reduction in UV absorbance may lead to the formation of toxic byproducts and to ensure the safety and functionality of photostabilized sunscreens.

### 2.5 Antioxidant Activity

**FIGURE 8:**
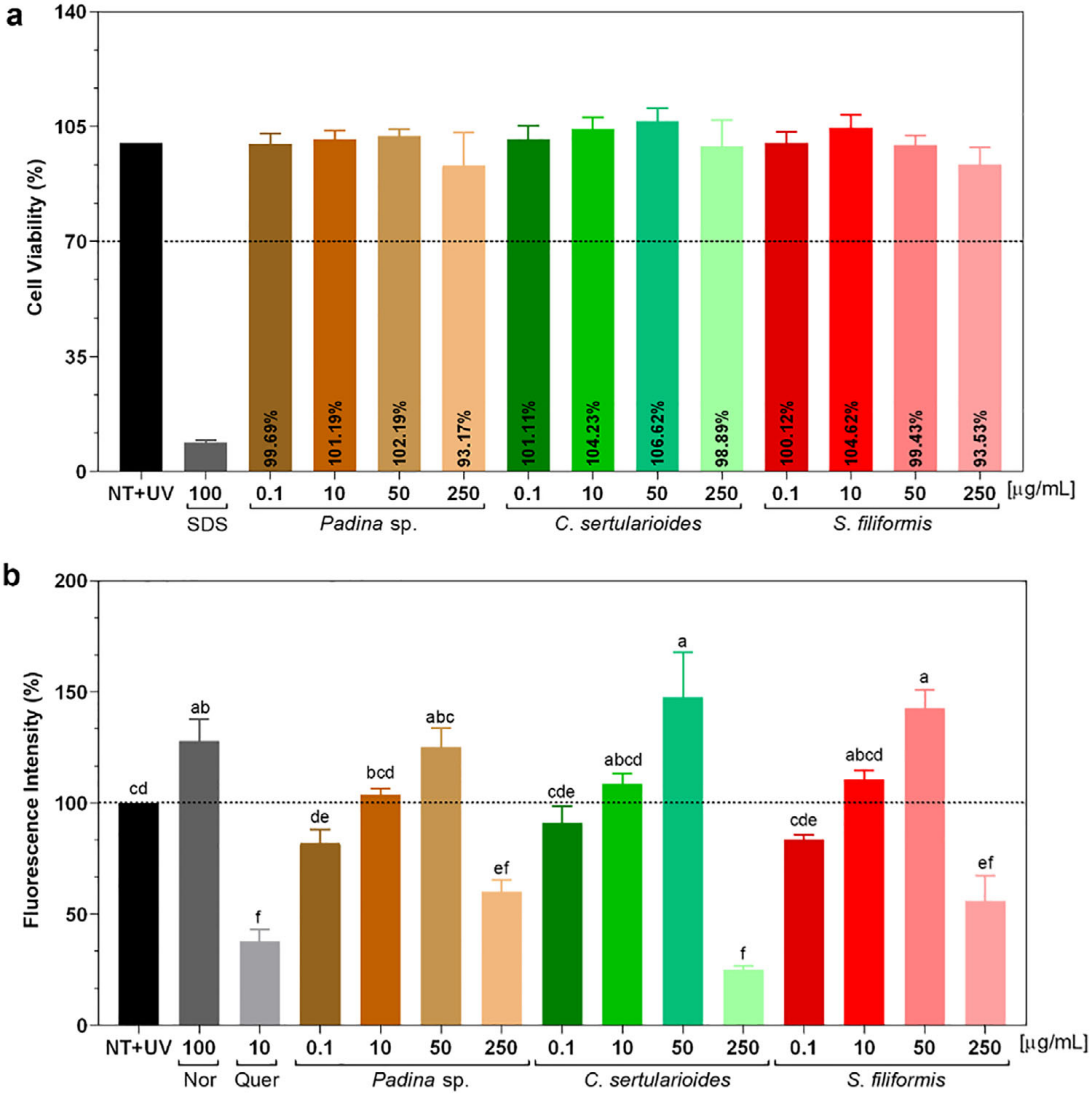
(a) Cell viability (%) of different concentrations (µg/mL) of *Padina* sp., *Caulerpa sertularioides*, and *Solieria filiformis* extracts; (b) protection against ROS generation in HaCat keratinocytes after UVA irradiation (4 J/cm^2^) using a fluorescent probe DCFH_2_‐DA. The results are expressed in fluorescence (%). Data are shown as mean ± SEM (*n* = 3). One‐way ANOVA with Tukey post hoc test was used. Letters denote significant differences between treatments. NT: non‐treated and nonirradiated control; NT + UV: non‐treated irradiated control; SDS: sodium lauryl sulfate used as positive cytotoxic control; *Q*: quercetin used as inhibitor control; and NOR: norfloxacin used as ROS generator. *Source*: Image created by the authors.

To ensure that the antioxidant effect was not associated with cytotoxicity, cell viability was assessed after treatment with different extract concentrations. The results showed that viability remained above 70% compared to the non‐treated control (NT), confirming that the reduction in ROS was not due to cell damage or death, but rather to the radical‐scavenging activity of the extracts (Figure 8a) [80]. On the basis of this, the crude extracts of *Padina* sp., *C. sertularioides*, and *S. filiformis* extracts were subsequently evaluated for their ability to inhibit intracellular ROS induced by UVA irradiation.

At a concentration of 250 µg/mL, *Padina* sp. reduced ROS levels by approximately 42%, a reduction that was statistically different from the non‐treated irradiated control (NT + UV) (*p* < 0.01) and similar to the positive control, quercetin (*p* < 0.05). GC–MS analysis revealed the presence of bioactive compounds with antioxidant potential, such as fucoxanthin, gallic acid, fucosterol, and octadecanol, whereas the ^1^H NMR spectrum detected characteristic signals of fatty acids and mannitol, in addition to low intensity of phenolics, such as phlorotannins [28, 57, 78]. The latter are typically abundant in brown algae, but their limited detection may be related to the type of extraction adopted, as also observed by Nour et al. [29]. Thus, the spectroscopic, chromatographic, and biological findings complement each other and converge to position *Padina* sp. extract as a promising natural source.

*C. sertularioides* extract showed a 75% reduction in free radical scavenging activity, statistically equivalent to that observed for quercetin (*p* > 0.01), as illustrated in Figure 8b. This significant effect can be attributed, initially, to the phenolics and flavonoids identified in *C. sertularioides*; they exert relevant antioxidant effects, as reported by Anjali et al. [62], in which they observed high rates of DPPH (89.7%) and ABTS (85.4%) radical scavenging, in addition to a high FRAP value (1027.36 ± 11.49 µmol Fe^2^⁺/g), mainly attributed to the presence of sulfated polysaccharides, with additional contributions from phenolic compounds. In addition, the pigments such as chlorophylls and carotenoids present and abundant in green algae, also play a role in neutralizing ROS by absorbing UV radiation, as observed in the present study [60, 86, 87]. These findings are compatible with the spectral data obtained, which indicate the presence of antioxidant metabolites through GC–MS and ^1^H NMR. Thus, the fractionation and isolation of the active constituents can enhance the observed biological effects.

At a concentration of 250 µg/mL, *S. filiformis* reduced ROS levels by approximately 42%, which resulted in a statistically significant difference compared to the untreated irradiated control (NT + UV) (*p* < 0.01) and was also similar to the positive control, quercetin (*p* < 0.05). This result can be attributed to its chemical composition, evidenced by GC–MS and ^1^H NMR analyses. Fatty acids such as palmitic, oleic, and arachidonic acids were identified [63]. Complementarily, ^1^H NMR spectra revealed signals compatible with bromophenols and glycosylated structures, such as MAAs and lectins, all associated with antioxidant and immunomodulatory properties [37, 70]. Sousa et al. [37] analyzed hydrolyzed extracts of *S. filiformis* and determined antioxidant activity with ABTS (1022.4 ± 42.3 µM TE) and FRAP (138.6 ± 6.8 µM TE).

The results, overall, demonstrate that the crude extracts of *Padina* sp., *C. sertularioides*, and *S. filiformis* possess distinct and complementary photoprotective and antioxidant properties. The ability to absorb UV radiation, particularly in the UVB range, combined with its ROS‐reducing effects, reinforces its potential as a natural bioactive ingredient in dermocosmetic formulations. Although all extracts were classified as photounstable under UVA exposure, their efficacy in mitigating UVA‐induced oxidative stress suggests that, when properly stabilized, they may serve as multifunctional agents for preventing acute and chronic photodamage. Given that UVA rays penetrate deeply into the skin, generate ROS, induce persistent DNA damage, and are closely associated with photoaging, hyperpigmentation, and cancer risk, increasing absorption in the UVA‐II and UVA‐I regions is essential [5]. State‐of‐the‐art sunscreens efficiently absorb part of UVA‐I, up to 370 nm, but not the longer UVA‐I, up to 400 nm. As this radiation range can cause deleterious effects, sunscreens must cover this entire wavelength to ensure effective photoprotection [88].

The three macroalgae presented complementary characteristics. Although *Padina* sp. exhibited broad UV–Vis absorption, with compounds such as fucoxanthin and fucosterol, it was the most unstable in UVA. *C. sertularioides* exhibited intense absorption and a high content of pigments and phenolic compounds, with high antioxidant effect, but a photodegradation similar to that observed for *Padina* sp. *S. filiformis* exhibited more discreet absorption but was the most stable to radiation and also maintained good antioxidant activity. These results suggest that combining the extracts can enhance the UV absorption spectrum, as well as can increase stability and enhance the antioxidant action in photoprotective formulations [79, 80].

### 2.6 Toxicity Assessment

Marine‐derived compounds intended for topical use require comprehensive photosafety evaluation, particularly when intended for exposure to sunlight. This section presents the results of the in vitro photosafety assays performed on crude extracts of *Padina* sp., *S. filiformis*, and *C. sertularioides*, including assessments of phototoxicity and irritation potential by short‐time exposure (STE) to evaluate toxicity potential.

### 2.7 Phototoxicity

Photosafety assessments are critical for evaluating the potential risks associated with compounds that are intended to be used in sunlight‐exposed skin. Certain compounds when exposed to UV radiation may undergo photoreactive processes that can compromise product efficacy and stability by producing toxic or inactive degradation products [7]. Consequently, exposure to these compounds can induce or worsen phototoxic or photoallergic responses in humans, such as erythema, sunburn, hyperpigmentation, and chronic skin damage [88].

Regulatory guidelines from agencies, such as the US Food and Drug Administration (FDA), European Medicines Agency (EMA), and the Organization for Economic Co‐operation and Development (OECD), require photosafety evaluations for both topically applied or systemically available substances, including cosmetics and pharmaceuticals. These guidelines are critical to ensure safety, stability, and efficacy during normal use conditions [89]. In the present study, the positive control, norfloxacin, presented mean photo effect (MPE) values within the recommended range of 0.3–0.9, confirming the validity of the assay [41]. The crude extracts of *Padina* sp., *C. sertularioide*s, and *S. filiformis* were considered phototoxic (Table 2 and Figure S3).

**TABLE 2:** Results of the neutral red uptake phototoxicity test in BALB/c 3T3 fibroblasts.

Samples	MPE		Prediction
*Padina* sp.	0.239	0.536	Phototoxic
*Caulerpa sertularioides*	0.697	0.567	Phototoxic
*Solieria filiformis*	0.375	0.571	Phototoxic
NOR	0.355	0.463	Phototoxic

*Note*: n  = 2, independent experiments.

Abbreviations: MPE, mean photo effect; NOR, norfloxacin.

The extracts of *Padina* sp., *C. sertularioides*, and *S. filiformis* exhibited phototoxic potential. However, these assays used complex crude extracts, and further studies should optimize the extraction process, fractionate bioactive‐rich fractions, and isolate key compounds, removing possible phototoxins from the studied fractions. Similar approaches have been successfully applied to marine‐derived microorganisms. Maciel et al. [90] evaluated fractions and isolated metabolites from the endophytic fungus *Annulohypoxylon stygium* associated with the seaweed *Bostrychia radicans*, identifying photostable and non‐phototoxic compounds with UVB‐absorbing properties after excluding phototoxic fractions. This strategy highlights the relevance of fractionation and targeted isolation to improve the safety and efficacy profile of potential photoprotective agents.

The 3T3 neutral red uptake (NRU) PT assay is highly sensitive and, because it lacks a skin barrier, directly exposes cells to all the sample constituents, which may overestimate the risk compared to models that simulate human skin, such as RHS models, that evaluated the stratum corneum (SC) penetration [80]. Tavares et al. [80], for example, reported that fucoxanthin, isolated from the algae *D. anceps*, exhibited phototoxicity potential in 3T3 fibroblast cultures (MPE = 0.917), but not in RHS models. The author attributed the result obtained to the fucoxanthin reduced bioavailability through the SC and in the stratified epidermis. The SC complexity restricts the penetration of molecules larger than 500 Da, and fucoxanthin, like other carotenoids, has a relatively large molecular structure, which can make it difficult to cross the skin's SC. Furthermore, viable epidermis is composed of keratinocytes, which are less sensitive than fibroblasts to both xenobiotics and UV radiation [91, 92].

GC–MS analysis provided a general chemical profile of the compounds present in the extracts, indicating substances with molecular masses below 500 Da, thus compatible with the criterion for skin penetration through the SC. However, skin penetration is not determined only by molecular weight; parameters such as lipophilicity, polarity, hydrogen bonding capacity, and molecular geometry also significantly influence this process [92]. Therefore, GC–MS was not effective for detecting volatile and semivolatile compounds of higher molecular weight, such as β‐carotene, lutein, astaxanthin, zeaxanthin, chlorophylls, phycobilins, and porphyrins, usually found in these algae extracts as well as responsible for their UV absorption range [66, 67].

### 2.8 Irritation Potential

The STE, as described in OECD Guideline No. 491, is an in vitro cytotoxicity‐based method developed to assess the ocular irritation potential of chemicals after brief contact with the corneal surface, without the use of animal testing. This assay uses a confluent monolayer of rabbit corneal cells from the Statens Seruminstitut (SIRC), which are exposed to the test substance for 5 min, followed by a 24‐h recovery period. Cell viability is then measured by the MTT assay, and substances that maintain viability above 70% are classified as nonirritating [42].

Crude extracts of *Padina* sp., *C. sertularioides*, and *S. filiformis* were initially prepared at a concentration of 250 µg/mL, based on prior evidence of antioxidant activity at this level, which supports its potential use in topical formulations. These solutions were subsequently tested at concentrations of 0.05% and 5% (w/v). At both concentrations, all extracts maintained cell viability above 70%, with values comparable to the untreated group (NT), used as the negative control (100%) (Figure S4). Consequently, on the basis of the STE classification criteria, all extracts were categorized as nonirritating at both concentrations tested. In contrast, the positive control (sodium dodecyl sulfate, SDS) significantly reduced cell viability to approximately 30%.

Although the STE test is traditionally used to assess eye irritation potential, there is a study that has explored modifications to this test protocol to expand its applicability to dermal toxicity assessment in the development of topical pharmaceuticals and cosmetic products [93, 94]. Mezaki et al. [93] evaluated the skin irritation potential of commercial skin lotions using an adapted in vitro STE approach. The modified protocol involved adjustments to both substance concentration and exposure duration. Notably, the results correlated strongly with those obtained from the reconstructed human epidermis (RHE) model, reinforcing the relevance of the STE method as a predictive tool for dermal irritation and highlighting its utility in preclinical screening before human patch testing. The author suggests that compared to the RHE model, which features a multilayered structure, the STE test is considered a more sensitive predictor of irritation. Therefore, based on animal testing data, substances that are nonirritating in the STE assay are generally also nonirritating in skin models with an SC.

However, limited information has been available on using the STE method to evaluate skin irritation. This study employed the STE method primarily as a cytotoxicity assay, with specific adaptations aimed at better simulating dermal exposure conditions. The findings suggest that the adapted STE test may be a useful tool in the preliminary assessment of the dermal toxicity of natural extracts. Its simplicity, sensitivity, and compatibility with high‐throughput screenings make it a promising strategy, especially when integrated with other complementary methodologies [94]. However, its use alone in the context of skin irritation assessment is still exploratory, and further validation with different classes of substances and formulations is needed to confirm its reproducibility and predictive value. Thus, STE should be considered a relevant complementary approach, but not a substitute, to the more established in vitro models.

## 3 Conclusion

In conclusion, this study provided a comprehensive chemical and biological characterization of *Padina* sp., *C. sertularioides*, and *S. filiformis*, tropical macroalgae from the coast of Bahia, Brazil. An integrative metabolomics approach combining GC–MS, ^1^H NMR spectroscopy, chemometric analyses (PCA, PLS‐DA), and molecular networking revealed distinct species‐specific profiles. *Padina* sp. was enriched in fucosterol, *C. sertularioides* in β‐sitosterol, and *S. filiformis* in cholesterol. Fatty alcohols were predominant in all species, although *S. filiformis* exhibited a more limited chemical fingerprint compared to the others. Multivariate analysis confirmed chemical clustering by phylum, with 80% classification accuracy and VIP markers supporting metabolite class discrimination. All extracts showed broad‐spectrum UV–Vis absorption, particularly in the UVA range (320–400 nm). *C. sertularioides* reduced intracellular ROS formation by 75%, followed by *Padina* sp. and *S. filiformis*, both with a 42% reduction at 250 µg/mL. However, photostability assays indicated degradation rates of ∼72% (UVB) and ∼47% (UVA) for *Padina* sp. and *C. sertularioides*, and 60% (UVB) and 35% (UVA) for *S. filiformis*. Regarding toxicity potential, all extracts were classified as nonirritating in the STE assay (cell viability >70% at 0.05% and 5%) but exhibited phototoxic potential. As these assays used complex crude extracts, further studies should optimize the extraction process, fractionate bioactive‐rich fractions, and isolate key compounds. This work represents an initial step in the biotechnological characterization of algae from the region, and the results reinforce the potential of macroalgae from the northeastern coast of Brazil as sustainable sources of functional compounds for topical applications, particularly in the development of green and reef‐safe cosmetic innovations.

## 4 Experimental Section

### 4.1 Algal Samples

The analyzed macroalgae were collected in Vilas do Atlântico—R. Praia de Grumari, Lauro de Freitas, Bahia, Brazil (12°89′54.1″ S, 38°29′43.0″ W), a coastal region, in 2023 and 2024. Taxonomic identification was performed by Dr. José Marcos de Castro Nunes (UFBA), a specialist in phycology, based on macro‐ and micromorphological characters, comparison with reference herbarium specimens, and consultation with standard taxonomic literature. The species are quite common and well known and present well‐defined morphological diacritics, allowing correct identification. The nomenclature of all species was cross‐referenced with the AlgaeBase database to ensure updated classification. The nomenclature was cross‐referenced with the AlgaeBase database to ensure updated classification. Voucher specimens representing all collected species were deposited in the Alexandre Leal Costa Herbarium (https://alcbufba.wixsite.com/meusite/algas) of the Institute of Biology of the Federal University of Bahia (UFBA, Salvador, Brazil) under the following codes: ALCB 149460 (*Padina* sp.), ALCB 149454 (*C. sertularioides*), and ALCB 149458 (*S. filiformis*). These records ensure taxonomic authentication, traceability, and reproducibility of the material used. Diagnostic images of representative specimens are provided in Figure S5. The morphological identification, combined with voucher deposition, provided a robust authentication of the algal material. Authorization to collect the material was obtained from the Ministry of the Environment (MMA, authentication code: 0881250120230420), and access to the genetic heritage was registered in the National System for the Management of Genetic Heritage and Associated Traditional Knowledge (SisGen, registration code: AB4CB0F).

### 4.2 Extract Preparation

The samples were maintained under controlled freezing conditions until subsequent experimental analyses. The extracts were obtained using the method with the necessary adaptations [80]. After thawing and cleaning, the algae were dehydrated in a forced‐air oven at 37°C for 72 h and then pulverized. For the extraction process, 2 g of each sample was weighed, and sequential extractions were performed with ethyl acetate (2 × 100 mL) and methanol (3 × 100 mL), with each solvent remaining in contact with the sample for 30 min under constant agitation at room temperature (approximately 25°C). The total extraction time was 150 min per sample, carried out in triplicate. At the end of the process, the extracts were concentrated using a rotary evaporator under low pressure, resulting in the final dried extracts.

### 4.3 Chemical Characterization

#### 4.3.1 GC–MS Analysis

The analysis of algae extracts was performed according to the methodology described by Santos et al. [95], with adaptations. An Agilent 7890B GC system coupled to a 5977C MSD mass selective detector (Agilent Technologies, Santa Clara, CA, USA) was used, with the following parameters: injection volume of 1 µL, in split mode (1:10); injector temperature, 260°C; DB‐5 chromatographic column (30 m × 0.25 mm × 0.25 µm); helium carrier gas, with a constant flow rate of 1 mL/min. The temperature program was started at 60°C, with a heating ramp of 4°C/min to 320°C, totaling 75 min of run. The interface was maintained at 260°C and the ion source at 230°C. The acquisition mode was full scan in the 50–650 *m/z* range, using electron impact ionization (EI) at 70 eV. Mass spectra were initially annotated by comparison with the NIST11 library. The raw data were converted to  .cdf format and uploaded to the GNPS platform (https://gnps.ucsd.edu) for deconvolution and subsequent construction of molecular networks. The networks were visualized using Cytoscape software (v3.9.1), and nodes were clustered based on spectral similarity, allowing the grouping of structurally related compounds. Retention times (RTs) were obtained directly from the chromatographic runs, as provided by the instrument. Metabolites were characterized by a comparison of both RT/RI values with the NIST11 mass spectral library and literature‐reported RI values. The details are summarized in Table S1, ensuring transparency and reproducibility of the chemical annotations. It should be noted that GC–MS annotations are putative, as they rely on spectral similarity (RI values, NIST library matches, and GNPS networking) [40].

#### 4.3.2 ^1^H NMR Analysis

For ^1^H NMR analysis, 5 mg of each extract was dissolved in 650 µL of deuterated dimethyl sulfoxide DMSO‐d_6_ (Sigma‐Aldrich) and transferred to 5 mm NMR tubes. Spectra were acquired on a Bruker Avance III spectrometer (400 MHz, 9.4 T) with 16 scans, applying selective solvent suppression (*δ* = 2.5 ppm) using presaturation. The data were processed using MestReNova software (v.14.1.2), including baseline correction, Gaussian apodization, and normalization to the maximum peak intensity. The processed spectra were exported for statistical analysis and compared with literature‐based metabolite databases, focusing on the identification of characteristic signals [96].

#### 4.3.3 Data Integration and Multivariate Statistical Analysis

The GC–MS and ^1^H NMR datasets were analyzed to characterize both volatile and nonvolatile metabolites present in the extracts. GC–MS data were interpreted via molecular networks generated on the GNPS platform and visualized in Cytoscape, whereas NMR spectra were processed and analyzed using MestReNova. Subsequently, the results from both approaches were combined to discuss the chemical similarities among the studied species. For statistical evaluation, the raw data were structured and processed with MetaboAnalyst 5.0 (https://www.metaboanalyst.ca/), applying PCA, PLS‐DA, and heatmap construction. Variables with a VIP > 1 and *p* < 0.05 (analysis of variance [ANOVA] with FDR correction) were considered significant for identifying potential bioactivity‐related biomarkers [40, 96].

### 4.4 Evaluation of Photoprotective Properties

#### 4.4.1 UV Absorption Spectra

The macroalgae extracts UV and visible‐light absorbance (280–700 nm) was determined by using a spectrophotometer Cary 60 UV–Vis (Agilent Technologies). The samples were dissolved in methanol at a concentration of 100 µg/mL, and absorbance readings were performed in triplicate. The critical wavelength (*λ*_c_) was calculated as the wavelength at which 90% of the total area under the absorbance curve, within the 280–400 nm range, is accumulated. This parameter was used to characterize the spectral distribution of the extracts in the UVA/UVB range [15, 40].

#### 4.4.2 Photostability Assessment

The samples were dissolved in methanol to obtain a final concentration of 100 µg/mL. Then, 1 mL of each sample solution was transferred to 10 mL beakers, and the solvent was evaporated using compressed air. The samples were subsequently irradiated with a UVA dose of 9 J/cm^2^, using irradiance emitted by a UVA lamp (Actinic BL/10#, Philips). After irradiation, the samples were redissolved in 1 mL of methanol, and the absorption spectra were obtained using a spectrophotometer (8453 UV–Vis—Agilent Technologies). The photostability test was performed in triplicate. The photostability of the samples was assessed by calculating the area under the absorption curve (AUC) within the UVB (280–320 nm) and UVA (320–400 nm) ranges, using the integration function of the MicroCal OriginPro software (version 8 SRO, OriginLab Corporation, Northampton, MA, USA). Photostability results were expressed as the percentage of the irradiated sample's AUC relative to that of the nonirradiated control, which was considered 100% [16, 80].

#### 4.4.3 Antioxidant Activity

The quantification of intracellular ROS in human immortalized keratinocytes (HaCaT, RRID: CVCL_0038), provided by the Cell Bank of Rio de Janeiro (BCRJ code 0341, Rio de Janeiro, Brazil), was performed after exposure to UVA radiation, using the 2′,7′‐dichlorodihydrofluorescein diacetate (DCFH_2_‐DA) probe, to evaluate the extracts antioxidant potential. After entering the cell due to its membrane permeability, the probe is hydrolyzed by intracellular esterases to form the nonfluorescent compound DCFH. In the presence of ROS, such as hydrogen peroxide, hydroxyl radicals, carbonate, and nitrite, DCFH is oxidized to dichlorofluorescein (DCF), a highly fluorescent molecule. This conversion is further enhanced under UV radiation, which promotes ROS generation. Therefore, the resulting fluorescence intensity of DCF is directly proportional to the intracellular ROS levels [80, 97]. A cell viability assay was initially performed to determine whether the fluorescence detected in the ROS assay was influenced by cell death rather than reflecting antioxidant activity. The keratinocytes were provided by the Cell Bank of Rio de Janeiro (BCRJ code 0341, Rio de Janeiro, Brazil). The cells were seeded in 96‐well plates at a density of 1 × 10^5^ cells/well and incubated for 24 h at 37°C in a 5% CO_2_ atmosphere. The cells were treated with the algae extract concentrations of 0.1, 10, 50, and 250 µg/mL. The cells were incubated for 1 h, washed, and maintained overnight at 37°C in a 5% CO_2_ atmosphere. First, the cell viability was determined by using the NRU assay. The positive cytotoxic control used was sodium dodecyl sulfate (SDS) (100 µg/mL). The absorbance of untreated cells was considered 100% to calculate the percentage of cell viability relative to the samples [16]. They were then treated with the algae extracts and controls and maintained incubated for 1 h. The substances quercetin and norfloxacin, at concentrations of 10 and 100 µg/mL, were used as ROS inhibitor and generator controls, respectively. After washing, the DCFH_2_‐DA solution was added, and the plates were incubated again before being exposed to UVA radiation with a total dose of 4 J/cm^2^. Fluorescence was measured using a microplate reader (BioTek Synergy HT, Winooski, VT, USA) with an excitation wavelength of 485 nm and an emission wavelength of 528 nm. The fluorescence of the untreated irradiated cells was considered 100% to calculate the relative percentage of ROS generation in treated cells [15, 16]. This analysis was based on three independent experiments, each carried out in triplicate. The resulting data were statistically analyzed using ANOVA, a parametric test, followed by Tukey's post hoc test for multiple comparisons.

#### 4.4.4 Phototoxicity and Safety Evaluation

##### 4.4.4.1 Phototoxicity

Phototoxicity was evaluated based on cell viability in the presence and absence of UVA radiation, according to the neutral red (Merck, Darmstadt, Germany) uptake test using 3T3 fibroblasts according to OECD No. 432 guideline [41]. Mouse embryonic fibroblast cell line (BALB/c 3T3 clone A31, RRID: CVCL_0184), obtained from the Cell Bank of Rio de Janeiro (BCRJ code 0047, Rio de Janeiro, Brazil). Initially, they were suspended in supplemented DMEM, seeded in 96‐well plates at a density of 1 × 10^4^ cells/well, and incubated for 24 h. After this period, the plates were washed twice with phosphate buffered saline (PBS) and then treated with eight different concentrations (6.74–100 µg/mL) of the extracts (previously diluted in PBS containing 1% DMSO) and incubated for 1 h. Norfloxacin (Sigma‐Aldrich, St. Louis, MO, USA) was used as a positive control. One of the plates was irradiated with a total UVA dose of 9 J/cm^2^, provided by a solar simulator (SOL‐500 with a metal halide lamp and H1 filter, Dr. Honle AG, Planegg, Germany), whereas the other was kept in the dark. After exposure, both plates were washed, fresh culture medium was added, and the cells were incubated for another 24 h. Therefore, the cell viability was determined by incubating the cells for 3 h with a culture medium containing 50 µg/mL of the neutral red. This vital dye is taken up by viable cells and accumulates in their lysosomes, reflecting cell integrity [41]. After incubation, the cells were washed with PBS to remove dye excess. Desorption of the incorporated dye was performed using a solution composed of water, ethanol, and acetic acid (49%:50%:1%), and the absorbance was measured at 540 nm using a microplate reader (BioTek Synergy HT, Winooski, VT, USA). Viable cells accumulate the dye in their lysosomes, resulting in higher absorbance, whereas damaged or dead cells show reduced dye uptake, indicating loss of membrane integrity. The assay was performed in two independent experiments. Data analysis was conducted using Phototox Software 2.0, which calculated the MPE. MPE values were interpreted according to OECD criteria: MPE < 0.10 indicates no phototoxicity; 0.10 ≤ MPE < 0.15 indicates probable phototoxicity; and MPE ≥ 0.15 indicates phototoxicity [41].

##### 4.4.4.2 Irritation Potential

The irritation potential was evaluated using the rabbit corneal cell line (SIRC—Statens Seruminstitut Rabbit Cornea, RRID: CVCL_2724), obtained from the Cell Bank of Rio de Janeiro (BCRJ code 0224, Rio de Janeiro, Brazil). The STE in vitro assay was adapted to evaluate the cytotoxicity of the extracts, following OECD Guideline No. 491, an alternative method to animal testing [42]. The SIRC cells were seeded in 96‐well plates. After incubation, the extracts, prepared in 0.05% and 5% solutions of 250 µg/mL concentration, were applied to the wells in triplicate for 5 min at room temperature. The cells were then incubated with a 3‐(4,5‐dimethylthiazol‐2‐yl)‐2,5‐diphenyltetrazolium bromide (MTT) solution for 2 h at 37°C. The MTT assay is a colorimetric method that measures cell viability based on mitochondrial activity. In viable cells, mitochondrial enzymes reduce the yellow MTT salt to purple formazan crystals, which accumulate inside the cells. After removal of the MTT solution, hydrochloric acid in isopropanol was added to extract the MTT. Absorbance was measured using a microplate reader (BioTek Synergy HT) at 570 nm. Cell viability was calculated by subtracting absorbance values from the blank, and samples were evaluated on the basis of the remaining cell concentration. According to the prediction model described in OECD Guideline No. 491, samples with a mean viability ≤70% were classified as Category 1 (irritant), indicating severe and irreversible ocular damage, whereas those with viability >70% were classified as no category (nonirritant) [42].

## Author Contributions

**Keila Almeida Santana**: conceptualization, methodology, investigation, writing – original draft preparation. **Isadora de Jesus da Silva**: methodology, investigation, writing – review and editing. **Victor Pena Ribeiro**: supervision, funding acquisition, writing – review and editing. **José Marcos de Castro Nunes**: supervision, funding acquisition, writing – review and editing. **Hosana Maria Debonsi**: supervision, funding acquisition, writing – review and editing. **Ian Castro‐Gamboa**: supervision, funding acquisition, writing – review and editing. Lorena Rigo Gaspar: supervision, funding acquisition, writing – review and editing. **Gustavo Souza dos Santos**: supervision, project administration, conceptualization, writing – review and editing. **Aníbal de Freitas Santos Júnior**: resources, supervision, project administration, funding acquisition, conceptualization, writing – review and editing. All authors have read and agreed to the published version of the manuscript.

## Conflicts of Interest

The authors declare no conflicts of interest.

## Supporting information




**Supporting Information File 1**: cbdv70569‐sup‐0001‐SuppMat.pdf

## Data Availability

The data supporting the findings of this study are available from the corresponding author upon reasonable request.
