# Supplementary material for: Biotechnological Potential of Seaweeds from Bahia, Brazil: Metabolomic insights, Photoprotection and Antioxidant Activity
Source: Chem Biodivers. 2025 Oct 15;22(12):e02708. doi: 10.1002/cbdv.202502708 (PMC12715994; doi:10.1002/cbdv.202502708)

Supporting Information

Biotechnological Potential of Seaweeds from Bahia, Brazil: Metabolomic insights, Photoprotection and Antioxidant Activity

Keila Almeida Santana*[a], Isadora de Jesus da Silva, Victor Pena Ribeiro, José Marcos de Castro Nunes, Hosana Maria Debonsi, Ian Castro-Gamboa, Lorena Rigo Gaspar, Gustavo Souza dos Santos, Aníbal de Freitas Santos Júnior*.

[a] K. A. Santana, Dr. A. F. Santos Júnior. Department of Life Sciences. State University of Bahia (UNEB). Street Silveira Martins, 2555, Cabula, Salvador, BA 41150-000, Brazil. E-mail: keilaalmeidasantana@gmail.com; anibaljrr@uol.com.br

Table S1. Putative identification of metabolites detected by GC–MS in macroalgae extracts (MSI level 2). Retention times (RT), base peaks, match scores, cosine scores, and species distribution are provided.

Compound	MF	RT (min)	Base peak (m/z)	Match (%)	Cosine Score	Detected in species
2-n-Heptyfuran	$C_{11}H_{18}O$	30.50	81	75.1	0.88	*Padina* sp.
Tridecanoic acid	$C_{13}H_{26}O_2$	32.19	73	88.9	0.97	*S. filiformis*
n-Hexadecanoic acid	$C_{16}H_{32}O_2$	32.20	73	98.3	0.99	*Padina* sp., *S. filiformis*
Tetradecanoic acid	$C_{14}H_{28}O_2$	27.96	73	85.2	0.97	*S. filiformis*
Hexadecanol	$C_{16}H_{34}O$	30.54	83	80.3	0.89	*C. sertularioides*
Octadecanol	$C_{18}H_{38}O$	35.32	55	80.2	0.92	*Padina* sp., *C. sertularioides*
Oleic acid	$C_{18}H_{34}O_2$	35.31	69	85.8	0.97	*S. filiformis*
Arachidonic acid	$C_{20}H_{32}O_2$	38.18	79	83.6	0.97	*S. filiformis*
Cholesterol	$C_{27}H_{46}O$	49.99	50	89.7	0.96	*S. filiformis*
Fucosterol	$C_{29}H_{50}O$	52.50	414	96.6	0.63	*Padina* sp.
β-sitosterol	$C_{29}H_{50}O$	52.49	129	90.8	0.95	*C. sertularioides*

Figure S1. Multivariate analysis of the GC-MS chemical profiles of macroalgae extracts. **a.** PCA: Score plot revealing three distinct clusters. **b.** PLS-DA: Supervised model demonstrating a more pronounced separation between taxonomic groups. **c.** VIP Scores: Graph of the main metabolites responsible for discrimination between species, with a heat map indicating relative abundance (blue = low, red = high).

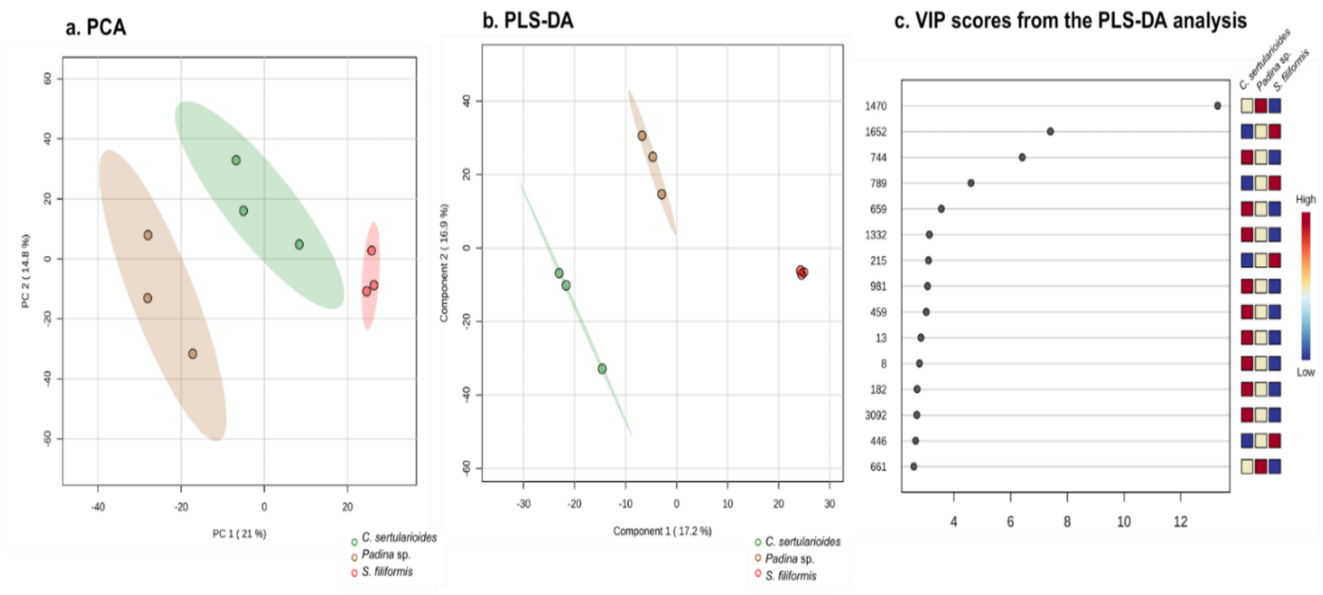

Figure S2. PLS-DA of ¹H NMR spectral data from macroalgae extracts. (a) Score plot showing the discrimination between species from different taxonomic phyla (*S. filiformis*, *Padina sp.,* and *C. sertularioides*), based on their chemical profiles. (b) VIP scores identify the most relevant spectral bins contributing to group separation.

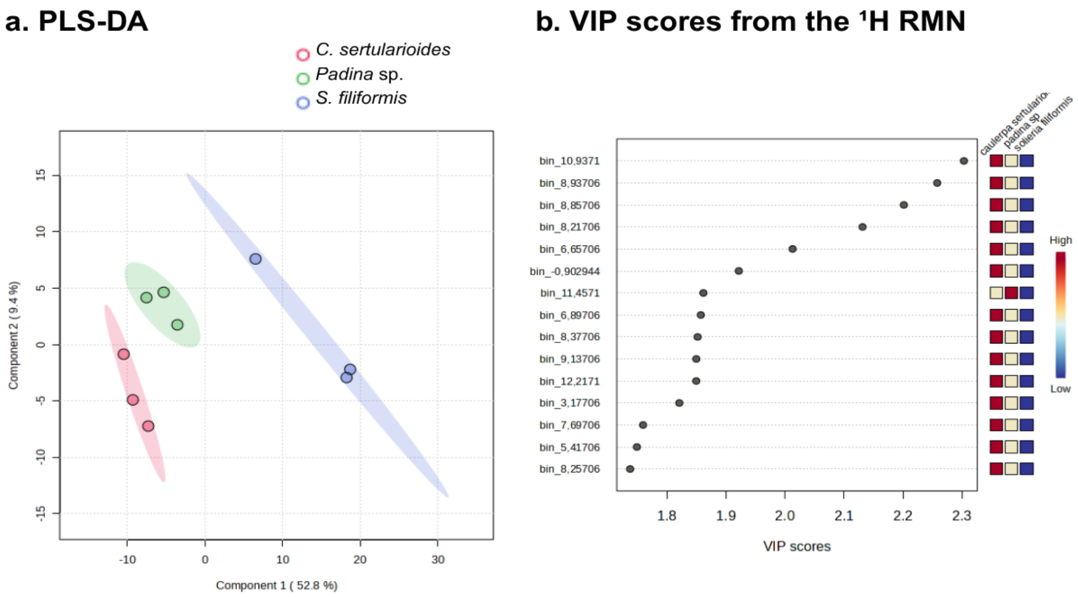

Figure S3. Dose-response curves of crude extracts of the Bahia macroalgae and control (a) *Padina* sp., (b) *C. sertularioides* (c) *S. filiformis* (d) NOR obtained by Neutral Red Uptake Phototoxicity Test in BALB/c 3T3 fibroblasts. The graphs were plotted using the Phototox 2.0 software program. The blue dots refer to non-irradiated cells (-UV) and the yellow dots to irradiated ones (+UV). The doses evaluated were: 6.81, 10, 14.7, 21.4, 31.6, 46.4, 68.1, and 100 µg.mL^{-1}. NOR: norfloxacin (positive control).

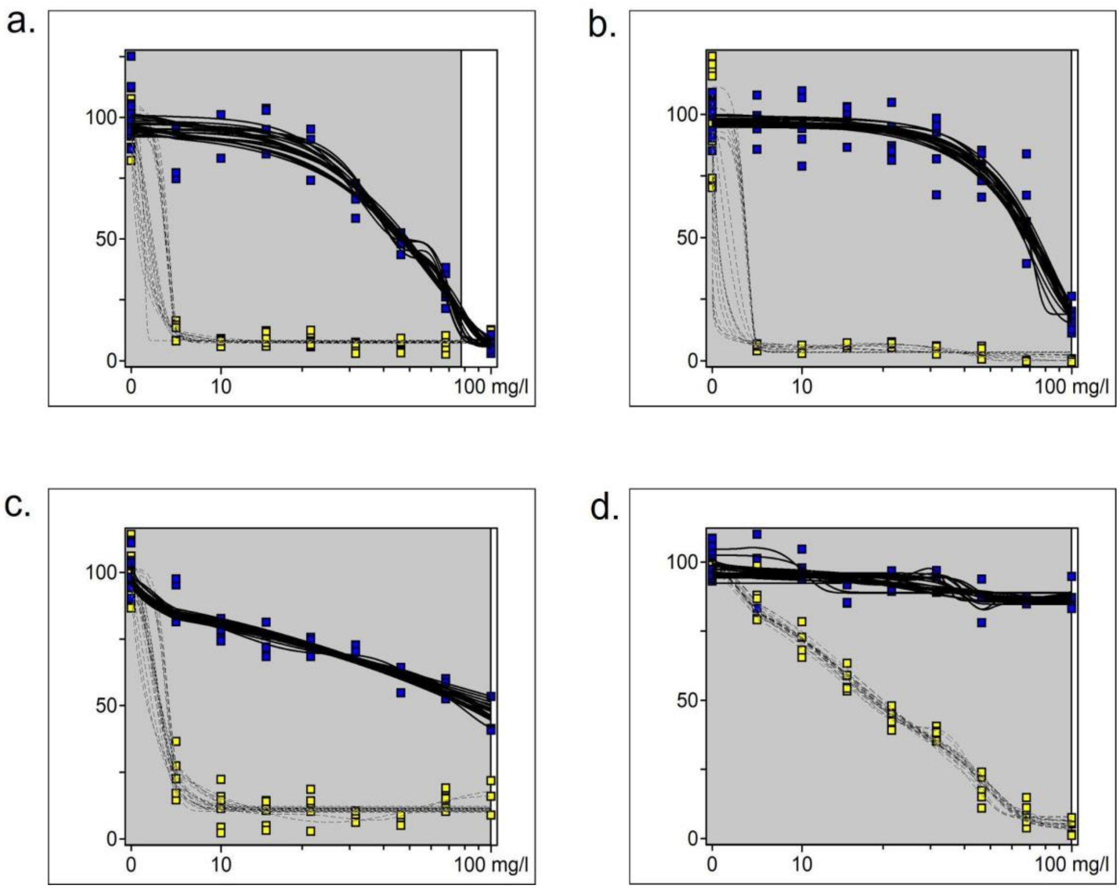

Figure S4. Cell viability results for STE performed for *Padina* sp*., C. sertularioides,* and *S. filiformis* extracts in 5% and 0.05% (n=3, independent experiments).

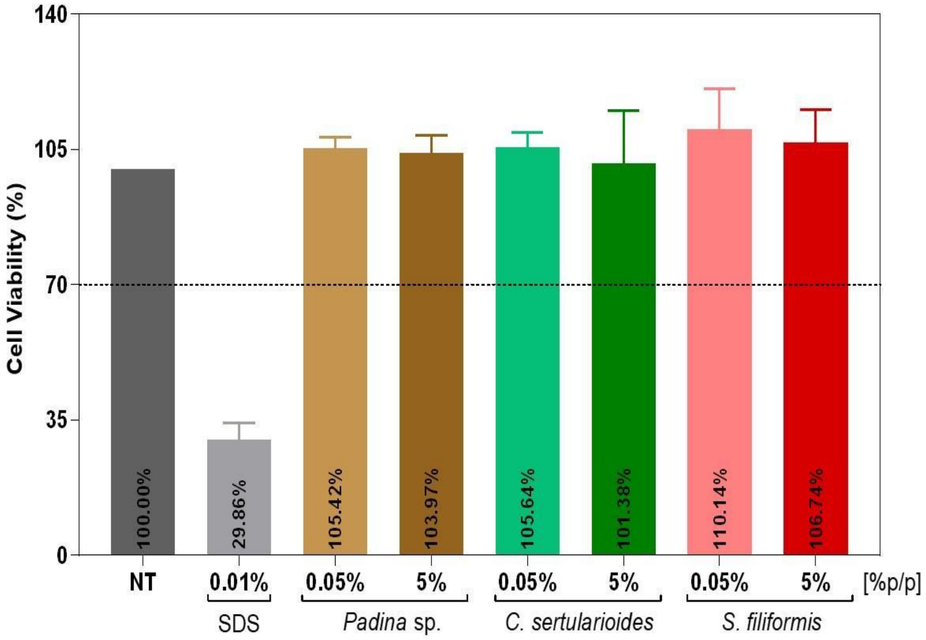

Figure S5. Representative field and microscopic images of the collected macroalgae species: (a) *Padina* sp. (Ochrophyta), (b) *Caulerpa sertularioides* (Chlorophyta), and (c) *Solieria filiformis* (Rhodophyta). Diagnostic morphological characters are highlighted, supporting species identification and voucher deposition at the Alexandre Leal Costa Herbarium (ALCB/UFBA).

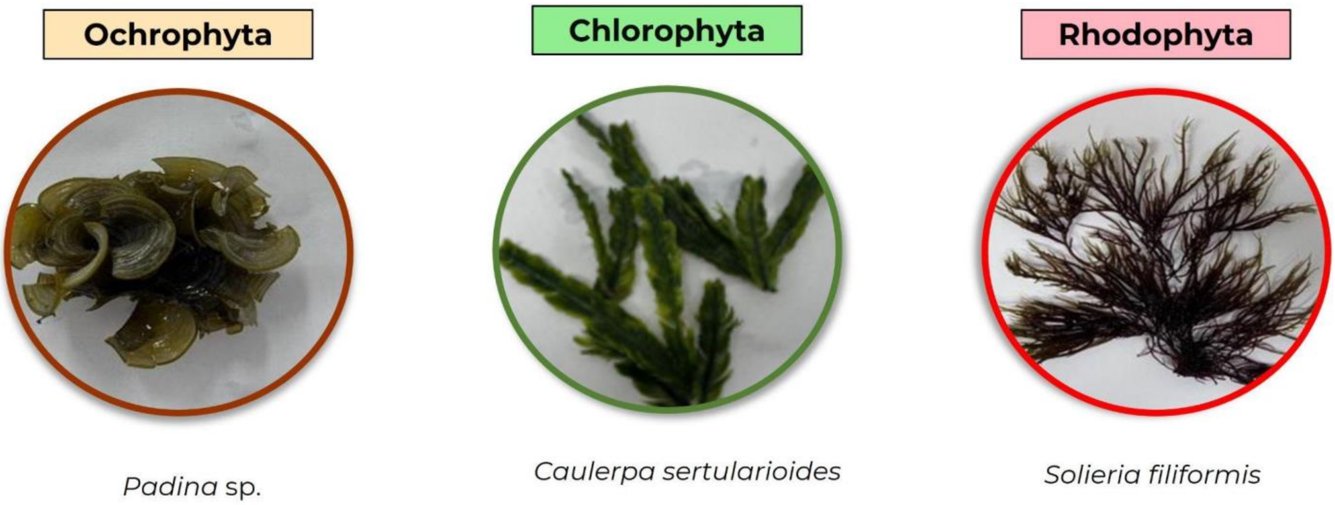

Supplement: Supplementary file 1 — Supporting Information File 1: cbdv70569‐sup‐0001‐SuppMat.pdf [file CBDV-22-e02708-s001.pdf]
